# Laser-Assisted Melt Electrospinning of Poly(L-lactide-co-ε-caprolactone): Analyses on Processing Behavior and Characteristics of Prepared Fibers

**DOI:** 10.3390/polym14122511

**Published:** 2022-06-20

**Authors:** Zongzi Hou, Haruki Kobayashi, Katsufumi Tanaka, Wataru Takarada, Takeshi Kikutani, Midori Takasaki

**Affiliations:** 1 Doctoral Program of Materials Chemistry, Graduate School of Science and Technology, Kyoto Institute of Technology, Matsugasaki, Sakyo-ku, Kyoto 606-8585, Japan; d9871502@edu.kit.ac.jp; 2 Faculty of Materials Science and Engineering, Kyoto Institute of Technology, Matsugasaki, Sakyo-ku, Kyoto 606-8585, Japan; haruki@kit.ac.jp (H.K.); ktanaka@kit.ac.jp (K.T.); 3 Department of Materials Science and Engineering, Tokyo Institute of Technology, 2-12-1 Ookayama, Meguro-ku, Tokyo 152-8550, Japan; takarada.w.aa@m.titech.ac.jp; 4 School of Materials and Chemical Technology, Tokyo Institute of Technology, 4259 Nagatsuta-cho, Midori-ku, Yokohama 226-8503, Japan; kikutani.t.aa@m.titech.ac.jp; 5 Center for Fiber and Textile Science, Kyoto Institute of Technology, Matsugasaki, Sakyo-ku, Kyoto 606-8585, Japan

**Keywords:** poly(L-lactide-co-ε-caprolactone), melt electrospinning, nanofibers, electrical force, birefringence, crystallinity, thermal properties, molecular orientation, crystalline structure

## Abstract

The laser-assisted melt electrospinning (LES) method was utilized for the preparation of poly(L-lactide-co-ε-caprolactone) (PLCL) fibers. During the process, a carbon dioxide laser was irradiated, and voltage was applied to the raw fiber of PLCL. In situ observation of fiber formation behavior revealed that only a single jet was formed from the swelling region under the conditions of low laser power and applied voltage and feeding rate, whereas multiple jets and shots were produced with increases in these parameters. The formation of multiple jets resulted in the preparation of thinner fibers, and under the optimum condition, an average fiber diameter of 0.77 μm and its coefficient of variation of 17% was achieved without the formation of shots. The estimation of tension and stress profiles in the spin-line was also carried out based on the result of in situ observation and the consideration that the forces originated from surface tension, electricity, air friction, and inertia. The higher peak values of tension and stress appearing near the apex of the swelling region corresponded to the formation of thinner fibers for the condition of single-jet ejection. Analyses of the molecular orientation and crystallization of as-spun fibers revealed the formation of a wide variation of higher order structure depending on the spinning conditions.

## 1. Introduction

In recent decades, the nanotechnology that has been developed has influenced every aspect of modern manufacturing systems. There is a large demand for employing nanofibers in a broad spectrum of applications, including high performance filtration/mat, biological and biomedical scaffolds, electronic devices because of a large surface/volume ratio, abundant pores between nanofibers, superior flexibilities, etc. [1,2,3,4,5].

Among the extensive range of nanotechnologies, electrospinning technology opens a door to readily produce ultrafine fibers with a nanoscale diameter and has evolved into one of the most economically and technically efficient methods to achieve nanofibers [6,7]. Generally, electrospinning could be divided into solution electrospinning and melt electrospinning. Considering the environmental friendliness and safety for in vivo application, melt electrospinning is a more preferred option due to the absence of residual toxic solvents in the air and nanofibers.

In conventional melt electrospinning, however, the long residence time of polymer melt, which is necessary for producing thinner nanofibers, causes thermal degradation [8]. The urgency of solving this problem drives us to develop a novel method to produce nanofibers. Therefore, we have been investigating a new type of melt electrospinning, i.e., laser-heated melt electrospinning (LES) with CO_2_ laser irradiation, to produce nanofibers [7]. This process allows us rapid heating of polymers and short residence time of molten polymers at higher temperatures to suppress thermal degradation. 

For the purpose of achieving nanofibers with desirable fiber diameter and structure, understanding the mechanism of fiber formation in melt electrospinning is of great significance to control processing parameters. In melt electrospinning, cone-jet mode, where the charged polymer melt forms a Taylor cone under applied voltage and flow rate, is considered as a more desirable mode to achieve the reproducibility of fiber production. 

In previous studies, researchers made great efforts to establish a model for the theoretical analysis of the fluid dynamics of the electrospinning process in an attempt to predict the experimentally obtained jet diameter, the associated jet velocity, and extension rate under various processing conditions including flow rate, applied voltage, heating temperature, and nozzle-collector distance. E.J. Hinch [9] established a slender body model to analyze the deformation of fluid drop in electric force through the determination of the electric field around the drop assuming that the shape of the elongated drop is slender and axisymmetric. Feng et al. [10] applied the slender body model in the analysis of the jet and proposed a modified version of the electric field equation that introduced a new parameter (electric current). Xu et al. [11] focused on the electrodynamics of fiber jets in melt electrospinning theoretically and experimentally. These previous theoretical models for melt electrospinning provided the foundation for the estimation of electrodynamics parameters, including surface charge density, the electric field, and normal and tangential stresses in the cone region. Finally, the fiber diameter was determined based on the fluid dynamics throughout the whole process [12,13,14,15]. It should be noted that, followed by the ejection of a stable jet from a Taylor cone, the whipping of the jet occurs. The whipping motion region has been addressed by Hohman et al. [13] and Reneker et al. [16,17]. Resolving the process with an uncertain boundary condition of surface charge density at the inlet, which at times causes an unphysical long-wave instability of the jet, is also an important issue. 

Poly(L-lactide-co-ε-caprolactone) (PLCL), i.e., copolymer of L-lactide and ε-caprolactone, has been found to be a promising material for medical applications because of the hydrolysable and biocompatible properties and a desirable range of mechanical properties. Solution electrospinning of PLCL nanofibers with abundant pores, proper diameter for cell growth, and sufficient flexibility and mechanical properties satisfy the requirements for tissue engineering applications [18,19,20]. However, solution electrospinning for the production of nanofibers is critical for any in vivo applications because of the existence of residual toxic solvents [8]. Currently, only a few researchers focused on the fabrication of nanofibers by melt electrospinning and none of them produced PLCL nanofibers by the LES.

The lack of research on melt electrospinning of PLCL to fabricate nanofibers and the large demand of PLCL nanofibers in biomedical application drove us to conduct research on the preparation of PLCL nanofibers with desirable fiber structure and properties by melt electrospinning. In order to fill the gap between research and demand, and to expand the application of PLCL in biomedical fields, in this study, influences of feeding rate and applied voltage on fiber jet formation was highlighted. Through the theoretical analysis of fiber thinning behavior and the estimation of forces/stresses of various origin acting in the cone region, the mechanism of fiber jet formation was further studied and clarified with the purpose of achieving PLCL nanofibers. Based on this result, the relationship between the fiber thinning behavior and the structure and properties of nanofibers in the PLCL webs, including fiber diameter, thermal properties, molecular orientation, and crystalline structure, prepared under various feeding rates and applied voltage were investigated.

## 2. Materials and Methods

### 2.1. Materials

The PLCL used in this research was a copolymer of L-lactide/ε-caprolactone with the molar ratio of 75/25 and the molecular weight of ca. 300,000 g/mol. A monofilament of PLCL prepared from this polymer was supplied by Kono Seisakusho Co., Ltd., Ichikawa, Japan, and used as a raw fiber for the LES. The raw fiber for LES had an average diameter of 191 μm and coefficients of variation (CV) of 3%.

### 2.2. Laser-Heated Melt Electrospinning Process (LES)

**Figure 1:**
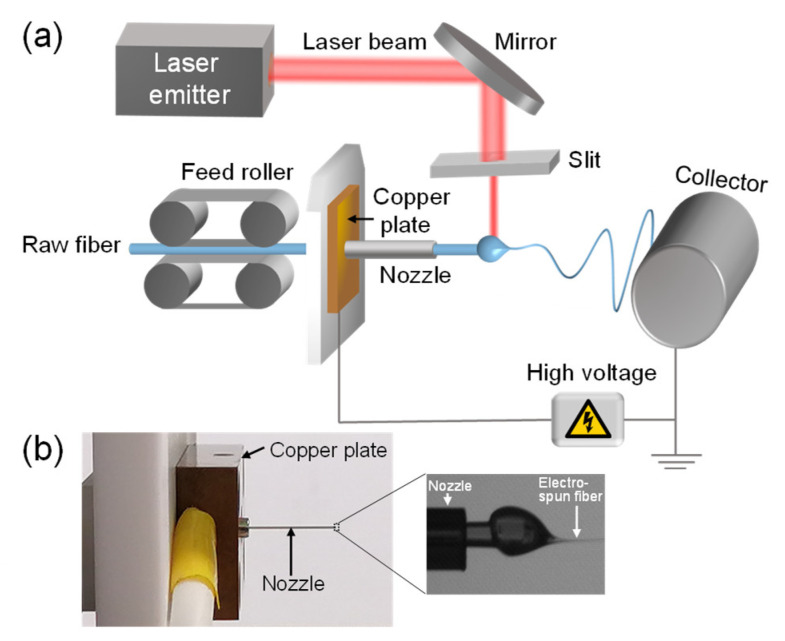
(**a**) Schematic diagram of laser-heated melt electrospinning (LES) process and (**b**) nozzle region of LES apparatus with enlarged photograph showing fiber thinning behavior.

As depicted in Figure 1, the LES system was composed of an electrospinning apparatus (NEU-010, Katotech Co., Ltd., Kyoto, Japan) and a heating apparatus with a CO_2_ laser irradiation system (wavelength: 10.6 μm, PIN-30R, Onizuka Glass Co., Ltd., Oume, Japan) [21]. The circular laser beam was deformed into an elliptical shape using a cylindrical lens and further narrowed by a cut-off slit, which is an alumina plate with a rectangular hole with short and long axes of approximately 0.5 and 4 mm, respectively. The long axis of the slit was perpendicular to the direction of fiber production. The distance between the laser beam axis and the tip of the nozzle for pushing out the raw fiber was fixed to approximately 0.8 mm.

In the LES process, the raw PLCL fiber was led to a stainless steel nozzle (26 G; inner diameter: 0.25 mm; outer diameter: 0.46 mm) by a feed roller and pushed out from the tip of the nozzle for CO_2_ laser irradiation heating. The nozzle was connected to a copper plate to apply high voltages. The raw fiber pushed out from the nozzle was melted by laser irradiation, formed a swelling region due to its surface tension, and subsequently stretched towards the stationary collector roller with the diameter of 100 mm and width of 100 mm due to the applied voltage. The collector was positioned 60 mm away from the tip of the nozzle.

The LES conditions are summarized in Table 1. The laser powers of 8 and 20 W were adopted. For the condition of the constant feeding rate of 28.8 mm/min, applied voltage was varied from 6 to 23 kV. On the other hand, for the constant applied voltage of 20 V, the feeding rate was varied from 17.0 to 72.2 mm/min. The electrospinning apparatus was installed in a box, and relative humidity inside of the box was maintained at approximately 28% by delivering dry air flow. The temperature in the box was approximately 20 °C.

For the purpose of the real-time observation of fiber thinning behavior in the vicinity of the nozzle during LES, a charge-coupled device (CCD) camera with a telecentric lens of 2× magnification was used. The camera was connected to a laptop computer, and video images were recorded at a rate of 15 fps. Still-images were extracted from the video images with an interval of 1 s for each LES condition. 

### 2.3. Analysis of Fiber Formation Behavior near the Nozzle during LES

#### 2.3.1. Profiles of Diameter, Running Speed, Strain Rate, and Residence Time of Fiber

Diameter profiles of the fibers near the nozzle were analyzed based on the images acquired for various spinning conditions. An image processing software (WinROOF 2015, MITANI Corp., Fukui, Japan) was used to determine the boundary of running fibers.

The rule of mass conservation in the melt spinning process of steady state can be expressed by Equation (1) [22]:

(1) $$W=\rho \frac{\pi D^{2}\left(x\right)}{4}v\left(x\right)$$

where x is the distance from the nozzle, and $D\left(x\right)$ and $v\left(x\right)$ are the fiber diameter and the fiber running speed, respectively, at each position along the spin-line. The throughput rate W and the density of fiber ρ were assumed to be constant.

By ignoring the change in fiber density along the spin-line, the fiber running speed and strain rate at each position of the spin-line were calculated from the obtained diameter profiles using Equations (2) and (3), respectively [22]:

(2) $$v\left(x\right)={\left(\frac{D_{0}}{D\left(x\right)}\right)}^{2}v_{0}$$

(3) $$\dot{\varepsilon }\left(x\right)=\frac{dv\left(x\right)}{dx}$$

where $D_{0}$ is the diameter of the raw fiber, $v_{0}$ is the fiber feeding rate, and $\dot{\varepsilon }\left(x\right)$ is the strain rate at each position of the spin-line.

The residence time of the polymer, i.e., the time the fiber stays in the spin-line after leaving the tip of the nozzle, was also calculated as a function of the distance from the nozzle using Equation (4):

(4) $$t\left(x\right)=\int_{0}^{x}\frac{dx}{v\left(x\right)}$$

#### 2.3.2. Electrical Stress Analysis of the Fiber near the Nozzle

Based on the diameter profile described in the previous section, the distributions of surface charge density $\sigma_{e}\left(x\right)$, electric field $E\left(x\right)$, normal stress $T_{n}\left(x\right)$, and tangential stress $T_{t}\left(x\right)$ were estimated as functions of the distance from the tip of the nozzle. As in the case of the diameter profile, analysis could be made for the spin-line in the vicinity of the nozzle where the stable straight jet could be observed.

During the electrospinning, electrostatic traction, which depends strongly on the surface charge density and electric field, plays a significant role in the formation and development of a jet. In previous studies [10,12], the charge conservation equation was proposed as shown below:

(5) $$I=\pi R^{2}\left(x\right)KE\left(x\right)+2\pi R\left(x\right)v\left(x\right)\sigma_{e}\left(x\right)$$

where the total current in the jet ejected from the Taylor cone and resultant fiber I, and the conductivity of the material K are constant. The first and second terms in the right-hand side of Equation (5) corresponds to the conduction current and the convection current, respectively. In this equation, the axial electric field in the spin-line $E\left(x\right)$, the radius of the jet (fiber) $R\left(x\right)$, and the surface charge density of the jet (fiber) $\sigma_{e}\left(x\right)$ vary as a function of the distance from the tip of the nozzle.

Assuming slender-body theory [9,10], electric field distribution along the spin-line was calculated by Equation (6):

(6) $$E\left(x\right)=E_{\infty }\left(x\right)-\left[\frac{1}{\varepsilon_{0}}\frac{d\left(R\left(x\right)\sigma_{e}\left(x\right)\right)}{dx}-\frac{\left(\frac{\varepsilon }{\varepsilon_{0}}-1\right)}{2}\frac{d^{2}\left(E\left(x\right)R^{2}\left(x\right)\right)}{dx^{2}}\right]\ln\frac{L_{fin}}{R\left(0\right)}\;$$

where *E*(*x*) is the variation of the electric field along the spin-line, and $\varepsilon_{0}$ and ε are the dielectric constants of the ambience and polymer.

In the analysis, the applied external field $E_{\infty }\left(x\right)$ was assumed to be spatially uniform, i.e., $E_{\infty }\left(x\right)=V/L_{fin}$ where V is the applied voltage, and $L_{fin}$ is the distance between the tip of the nozzle and collector.

Then, the total current I could be simply calculated using Equations (7) and (8) for x=0 [1]:

(7) $$I=2\pi R\left(0\right)v\left(0\right)\sigma_{e}\left(0\right)$$

(8) $$\sigma_{e}\left(0\right)=\varepsilon_{0}E\left(0\right)$$

where $R\left(0\right)$ is the initial fiber radius, and $v\left(0\right)$ is the feeding rate. Accordingly, variation of surface charge density along the spin-line could be estimated by Equation (9):

(9) $$\sigma_{e}\left(x\right)=\frac{I}{2\pi R\left(x\right)v\left(x\right)}$$

#### 2.3.3. Dielectric Constant and Conductivity

The dielectric constant of PLCL, which is necessary for the estimation of normal stress on the surface of the jet (fiber), as shown in Equation (10), was measured using a microwave molecular orientation analyzer (MOA-6020, Oji Scientific Instruments, Co., Ltd., Amagasaki-shi, Hyogo, Japan). Prior to the measurement, a circular PLCL film with a thickness of 135 μm and diameter of 3.5 cm was prepared by a hot press under the compression temperature and pressure of 180 °C and 60 MPa. In the analyzer, a polarized microwave was irradiated perpendicularly to the film sample attached to a sample holder, which was rotated in a cavity resonator. The resonance frequency was 19.4 GHz. Based on the perturbation theory, the average dielectric constant (*ε*) of PLCL was obtained from the dielectric constant pattern [23,24], which indicated a virtually isotropic structure in the film plane.

In the present study, the conductivity of PLCL is at a low level of 1 × 10^−9^ S/m [25] compared with the solutions used in solution electrospinning. Therefore, the conduction current was neglected, indicating that the charges distribute only on the surface of fiber in the LES. The detailed parameters with their units used for the analysis are summarized in Table 2.

#### 2.3.4. Analysis of the Spin-Line Tension and Stress

**Figure 2:**
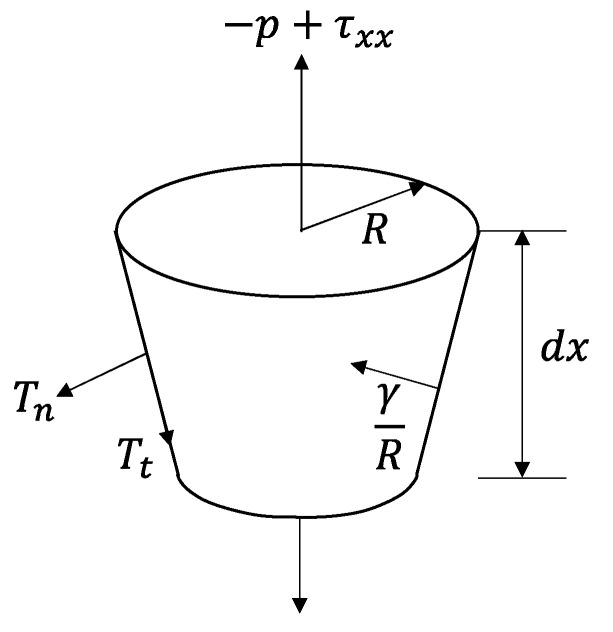
Momentum balance on a short section of a jet.

In addition, the momentum balance equation formulated by considering the forces on a short segment of the jet (Figure 2) [10,22] was applied to express the variations of tension and tensile stress along the spin-line:

(10) $$\frac{d}{dx}\left(\pi R^{2}\left(x\right)\rho v^{2}\left(x\right)\right)=\pi R^{2}\left(x\right)\rho g+\frac{d}{dx}\left[\pi R^{2}\left(x\right)\left(-p+\tau_{xx}\left(x\right)\right)\right]+\frac{\gamma }{R\left(x\right)}2\pi R\left(x\right)R^{\prime }\left(x\right)+2\pi R\left(x\right)\left(T_{n}\left(x\right)-T_{t}\left(x\right)R^{\prime }\left(x\right)\right)-2\pi R\left(x\right)\tau_{f}\left(x\right)$$

where g is the gravitational acceleration, $\tau_{xx}\left(x\right)$ is the axial viscous normal stress, p is the ambient pressure, γ is the surface tension (Table 2), $T_{n}\left(x\right)$ and $T_{t}\left(x\right)$ are the normal stress and tangential stress evolving on the surface of the jet (fiber) due to electricity (electric charge and electric field), and $\tau_{f}\left(x\right)$ is the shear stress on the surface of the jet (fiber) imposed by the air friction. The prime indicates the derivative with respect to x, and $R^{\prime }\left(x\right)$ is the slope of the jet surface.

The gravity term in Equation (10) (the first term on the right side) is negligible because LES is the horizontal process. The second term corresponds to the rheological force arising due to the elongational flow of the material. The third term, the cohesive force arising due to the surface free energy, was expressed by *γ*/*R*. The fourth term includes the normal stress $T_{n}\left(x\right)$ and tangential stress $T_{t}\left(x\right)$ evolving on the surface of the jet (fiber) due to electricity, which are determined by the surface charge density $\sigma_{e}\left(x\right)$ and electric field $E\left(x\right)$ and can be calculated by Equations (11) and (12) [10]:

(11) $$T_{n}\left(x\right)=\frac{\sigma_{e}{}^{2}\left(x\right)}{2\varepsilon_{0}}-\frac{\varepsilon_{0}-\varepsilon }{2}E^{2}\left(x\right)$$

(12) $$T_{t}\left(x\right)=\sigma_{e}\left(x\right)E_{t}\left(x\right)\;\approx \;\sigma_{e}\left(x\right)E\left(x\right)$$

The fifth term in Equation (10), the effect of air friction, was estimated based on the empirical equation for the relation between the Reynolds number and the air-friction coefficient of the spin-line [22].

Expressing each term in Equation (10), which related to the inertial force $F_{i}\left(x\right)$, rheological force $F\left(x\right)$, surface tension force $F_{s}\left(x\right)$, force caused by electric charge and electric field $F_{e}\left(x\right)$, and air-friction force $F_{a}\left(x\right)$, is as follows:

$$F_{i}\left(x\right)=\pi R^{2}\left(x\right)\rho v^{2}\left(x\right),\;F\left(x\right)=\pi R^{2}\left(x\right)\left(-p+\tau_{xx}\left(x\right)\right),\;\frac{dF_{s}\left(x\right)}{dx}=\frac{\gamma }{R\left(x\right)}\cdot 2\pi R\left(x\right)R^{\prime }\left(x\right),\;\frac{dF_{e}\left(x\right)}{dx}=2\pi R\left(x\right)\left(T_{n}\left(x\right)-T_{t}\left(x\right)R^{\prime }\left(x\right)\right),\;\;\mathrm{and}\;\frac{dF_{a}\left(x\right)}{dx}=2\pi R\left(x\right)\tau_{f}\left(x\right)$$

The differential type of force balance equation can be expressed by Equation (13):

(13) $$\frac{dF\left(x\right)}{dx}=\frac{dF_{i}\left(x\right)}{dx}-\frac{dF_{s}\left(x\right)}{dx}-\frac{dF_{e}\left(x\right)}{dx}+\frac{dF_{a}\left(x\right)}{dx}$$

Furthermore, variation of tension along the spin-line can be estimated by integrating each term in Equation (13) from distance 0 to *x* as shown in Equation (14). It should be noted that the initial tension is not readily known in this analysis:

(14) $$F\left(x\right)=F\left(0\right)+F_{i}\left(x\right)-F_{s}\left(x\right)-F_{e}\left(x\right)+F_{a}\left(x\right)$$

Additionally, the stress can be obtained by dividing each term in Equation (14) by the local cross-sectional area, $\pi R^{2}\left(x\right)$:

(15) $$\sigma \left(x\right)=\sigma \left(0\right)+\sigma_{i}\left(x\right)-\sigma_{s}\left(x\right)-\sigma_{e}\left(x\right)+\sigma_{a}\left(x\right)$$

#### 2.3.5. Fiber Temperature Measurement during LES

For the non-contact measurement of the temperature of the swelling region and jet in the melting zone of LES, an infrared radiation thermal imaging camera (InfReC H9000, Lens type: IRL-H9M15A, 839 × 632 pixels, Nippon Avionics Co., Ltd., Yokohama, Japan) was set 19.5 cm away from the fiber, which corresponded to the image resolution of 15 μm/pixel. The fiber temperature in the melting zone was estimated assuming the emissivity for PLCL is 0.95 [26,27].

### 2.4. Analysis of Fibers in the Web

#### 2.4.1. Scanning Electron Microscopy (SEM)

A scanning electron microscope (TM3000, Hitachi High-Tech Co., Tokyo, Japan) was applied for observation of electrospun PLCL fibers prepared at various LES conditions. The accelerating applied voltage was set to 15 kV. Prior to the SEM observation, a web sample was taped on a stage and overlaid with a layer of Au by ion sputter for 150 s. After obtaining the SEM images, the fiber diameter was analyzed through the measurement of a hundred points for each sample using an image processing application software (Image J version 1.8.0, U. S. National Institutes of Health, Bethesda, MD, USA).

#### 2.4.2. Differential Scanning Calorimetry (DSC)

DSC measurement of the electrospun fiber webs and the raw fiber were conducted using a differential scanning calorimeter (DSC 60A-Plus, Shimadzu Co., Kyoto, Japan). Prior to the measurement, approximately 1 mg of each electrospun web was weighed and sealed into an aluminum pan. The measurement was also conducted for the raw fiber sample (sample mass of approximately 5 mg) cut into a powder-like form. The feeding purged gas of dry nitrogen at a flow rate of 50 mL/min, the samples were heated at the heating rate of 10 °C/min. The temperature range for the measurement was from 0 to 225 °C.

The crystallinity ($X_{c}$ (%)) was calculated from Equation (16) [28]:

(16) $$X_{c}=\frac{\;\Delta H_{m}-\Delta H_{c}}{\Delta H_{m}^{0}\;LLA*}\times 100\;$$

where $\Delta H_{m}^{0}$ = 106 (J/g) is the enthalpy of fusion of PLLA crystals having the infinite crystal thickness [28], and $\Delta H_{m}$ and $\Delta H_{c}$ are the enthalpy of melting and cold crystallization, respectively. These peaks correspond to the melting and crystallization of the PLLA component, whereas *LLA*∗ is the L-lactide fraction in the copolymer, PLCL.

#### 2.4.3. Polarizing Microscope

In order to evaluate the molecular orientation of the raw PLCL fiber and the PLCL fibers in web samples, optical retardation was measured by a polarizing microscope (BX53-P, Olympus Co., Ltd., Tokyo, Japan) with insertions of a polarizing filter and a Bereck compensator. Prior to the observation, the raw PLCL fiber was cut diagonally to obtain the oblique cross-section with linearly changing thickness. Both raw PLCL fibers and web samples were placed between a glass slide and a cover glass to immerse in an immersion liquid. Birefringence was calculated using Equation (17):

(17) $$\Delta n=\frac{R}{d}$$

where Δn is the birefringence, R is the optical retardation, and d is the fiber diameter.

#### 2.4.4. Wide-Angle X-ray Diffraction (WAXD)

The wide-angle X-ray diffraction measurement was performed with Ni-filtered CuKα radiation of wavelength 0.154056 nm under applied voltage and current of 40 kV and 15 mA (Miniflex 600, Rigaku Co., Ltd., Tokyo, Japan) [21]. For the WAXD measurement, samples of the randomly oriented powder-like extremely short raw PLCL fiber and the stacked layers of several PLCL webs were prepared. Moreover, a bundle of the raw fiber was measured at the azimuthal angles of 0° and 90° for the investigation of the degree of orientation. The WAXD patterns were acquired by scanning a charge-coupled detector placed on the goniometer with the radius of 150 mm. The scanning was conducted within a Bragg angle (2θ) range from 5° to 30°, and at the scanning speed of 0.1°/min with a step size of 0.01°.

## 3. Results and Discussion

### 3.1. Analysis of Spinning Behavior

#### 3.1.1. Images of Spinning Behavior near the Nozzle during LES

**Figure 3:**
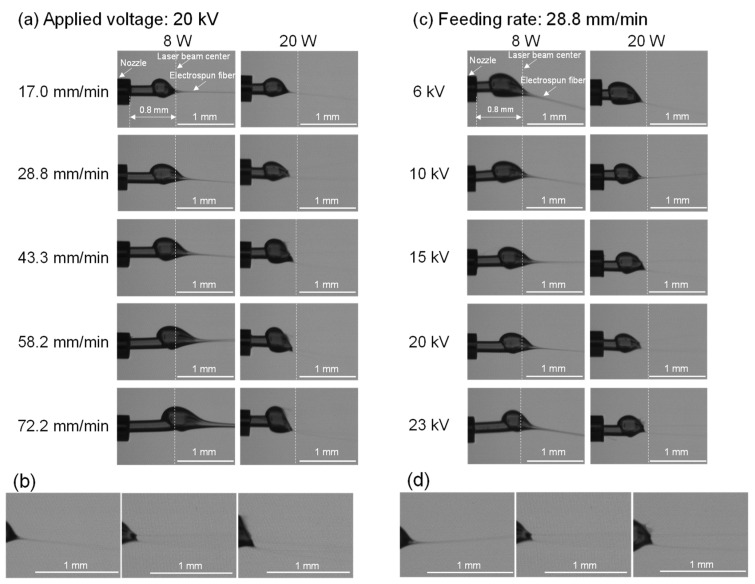
Photographs of fiber thinning behaviors near the nozzle: (**a**) effect of feeding rate at the applied voltage of 20 kV and laser power of 8 and 20 W, (**b**) enlarged photographs for conditions with the feeding rate of 17.0, 28.8, and 58.2 mm/min, applied voltage of 20 kV and laser power of 20 W, (**c**) effect of applied voltage at the feeding rate of 28.8 mm/min and laser power of 8 and 20 W, (**d**) enlarged photographs for conditions with applied voltages of 10, 20, and 23 kV, feeding rate of 28.8 mm/min, and laser power of 20 W.

Real-time observation of fiber thinning behavior in the vicinity of the nozzle was conducted during the LES of various conditions as shown in Figure 3. By the laser irradiation heating and applied voltage for the raw fiber, formation of a spherical droplet (swelling region), subsequent thinning of the droplet, and ejection of a jet to form fiber induced by the electrostatic force were observed. Figure 3a,c show the variations of thinning behavior with the changes of feeding rate and applied voltage, respectively. Enlarged images selected from (a) and (c) are shown in Figure 3b,d to distinguish the ejection of a single jet or multiple jets.

For investigating the effect of the feeding rate shown in Figure 3a, the applied voltage was set to 20 kV. At the laser power of 8 W, the swelling area became larger and prolonged downstream as the feeding rate increased from 17.0 to 72.2 mm/min. The shift of the swelling region to downstream with the feeding rate was also observed. From the images, it was noticed that only a single jet was ejected from the swelling region. The diameter of the ejected fiber increased with the increase in the feeding rate. This phenomenon may occur to fulfil the requirement that the volume of fiber fed into the swelling region needs to be in balance with that ejected from the region. A larger swelling region downstream may correspond to the necessity of effective laser irradiation heating for the higher feeding rate conditions. A similar result was reported for the laser heated melt electro spinning of sheet material by Shimada et al. [29].

On the other hand, at the laser power of 20 W, three types of fiber formation behaviors were observed with the increase in the feeding rate. At the feeding rate of 17.0 mm/min, a single jet was ejected from the swelling region, while multiple jets started to eject spontaneously when the feeding rate was increased to 28.8 mm/min. With a further increase in the feeding rate, shots and multiple jets started to form simultaneously from the swelling region. A similar phenomenon was reported in the research on the electrodynamic spraying of liquids by Hayati et al. [30]. They reported that the stable single jets are only produced with an optimal state of conducting liquid where an appropriate voltage is applied, while the multiple jets occur with the application of excessive electric potential.

It was speculated that a higher temperature at the laser power of 20 W led to the reduction in both viscosity and surface tension, where the effect of temperature was more significant for the reduction in viscosity than surface tension. Therefore, higher laser power caused Rayleigh instabilities with the increase in feeding rate at sufficiently high applied voltage of 20 kV, where excessive volume input into the swelling region yielded the ejection of multiple jets and shots instead of the formation of a thicker single jet as shown in Figure 3b.

For the effect of applied voltage shown in Figure 3c, the feeding rate was fixed at 28.8 mm/min. As applied voltage increased from 6 to 23 kV, at the laser power of 8 W, the swelling region contracted and shifted to downstream followed by the ejection of only a single jet from the tip of the swelling region. On the other hand, fiber thinning behavior was different at the laser power of 20 W. It was found that a single jet was ejected when the applied voltage was lower than 15 kV, whereas multiple jets were ejected spontaneously from 15 to 20 kV. At 23 kV, multiple jets accompanied by shots were observed, as shown in Figure 3d.

In research on electrohydrodynamic atomization (EHDA) or spraying, the occurrence of multiple jet instability (similar to rim instability) has been reported to be caused by the application of sufficiently high voltage. With the increase in charge density and exceeding the Rayleigh limit, coulomb explosion occurs [31]. Similar research on EHDA were also conducted by Doshi and Reneker [32] and Ganan-Calvo et al. [33], and it was speculated that the formation of multiple jets is caused by the higher charge density and an action of repulsive forces.

**Figure 4:**
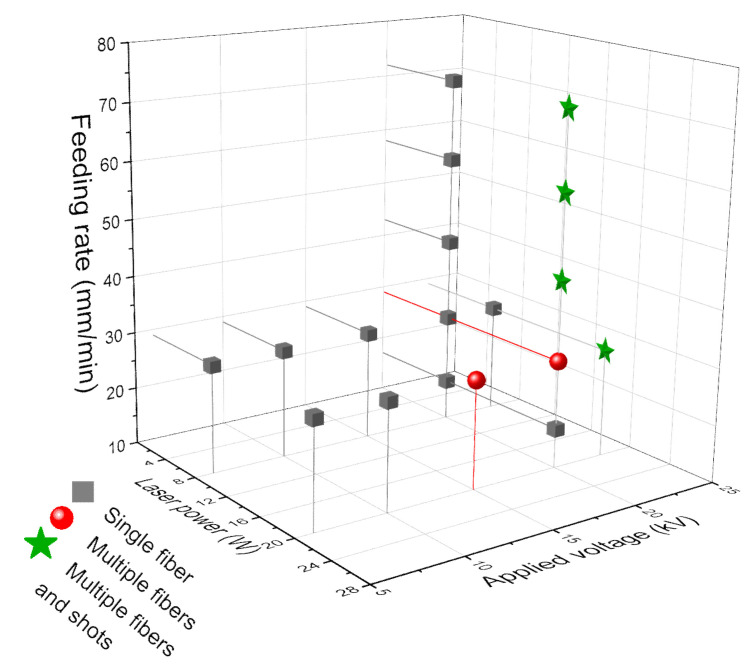
Categorization of fiber formation behaviors near nozzle varying with laser power, voltage and feeding rate.

The variation of LES behavior at different conditions is summarized in Figure 4, in which the fiber formation behavior was categorized into three types: i.e., formations of single jet (type I), multiple jets (type II), and multiple jets and shots (type III). It was observed that under the condition of relatively low laser power (8 W), applied voltage and feeding rate, only a single jet was prone to be formed, whereas, under the condition of relatively high laser power (20 W), applied voltage and feeding rate, multiple jets and shots tended to be produced.

#### 3.1.2. Fiber Temperature

**Figure 5:**
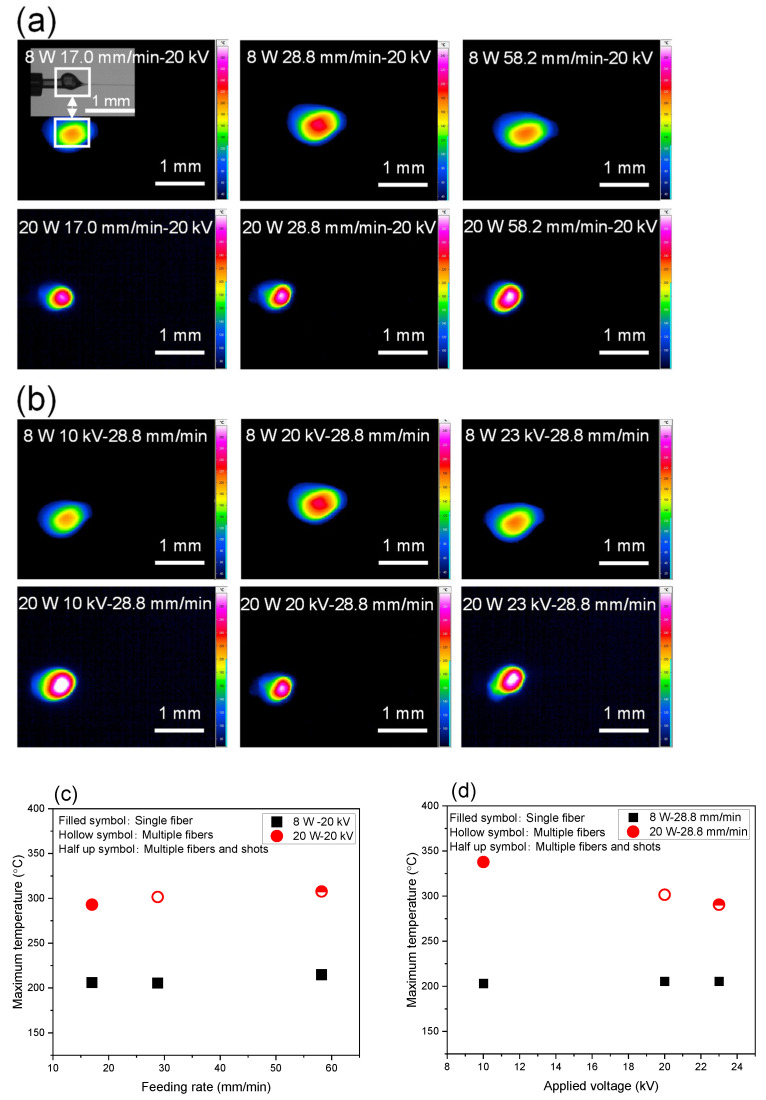
Thermography images of swelling region with laser power of 8 and 20 W; effects of (**a**) feeding rate and (**b**) applied voltage. Variations of maximum temperature with changes in (**c**) feeding rate, and (**d**) applied voltage at laser power of 8 and 20 W.

Infrared radiation thermography images of the swelling region for the LES conditions of varied feeding rate and applied voltage are shown in Figure 5a,b. Effects of feeding rate and applied voltage on maximum temperature obtained from the thermography images are shown in Figure 5c,d. For the constant applied voltage of 20 kV, with the increase of feeding rate, the high temperature area expanded and the maximum temperature in the swelling region increased from 206 to 216 °C, and from 293 to 308 °C for the laser powers of 8 and 20 W, respectively. On the other hand, for the constant feeding rate of 28.8 mm/min, increase in applied voltage resulted in little change in maximum fiber temperature in the swelling region at approximately of 205 °C for laser power of 8 W, while the increase in applied voltage led to the obvious monotonous decrease in the high temperature area and maximum temperature in the swelling region decreased from 338 to 293 °C at the laser power of 20 W.

Comparing the result of temperature analysis with the spinning behavior shown in Figure 3, it is interesting to note that, even though the shape and position of the swelling region were influenced significantly by the spinning conditions, maximum temperature of the swelling zone depended strongly on the laser power, while other spinning conditions at each laser power did not show significant influence on the relation between the heating and fiber thinning behaviors.

#### 3.1.3. Fiber Diameter Profiles

**Figure 6:**
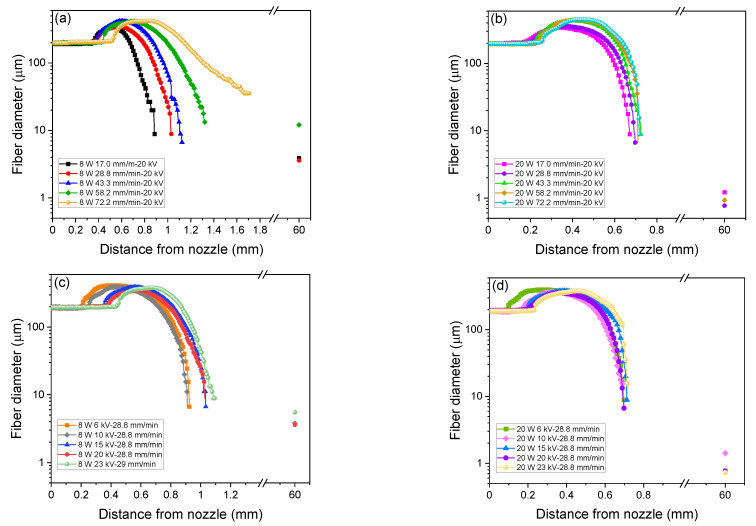
Effect of feeding rate on fiber diameter profiles for the applied voltage of 20 kV and laser power of (**a**) 8 W and (**b**) 20 W, and effect of applied voltage on fiber diameter profiles for the feeding rate of 28.8 mm/min and laser power of (**c**) 8 W and (**d**) 20 W. Data plotted at 60 mm are diameters analysed from the SEM images of the prepared web samples, which will be presented in Figure 7 and Figure 8.

**Figure 7:**
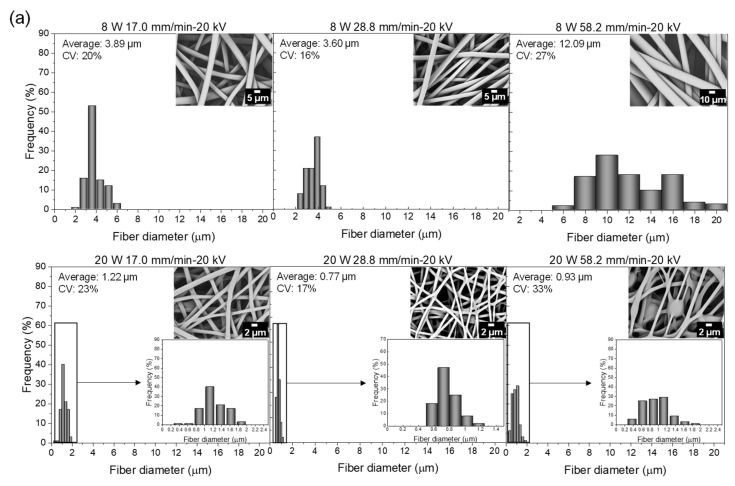

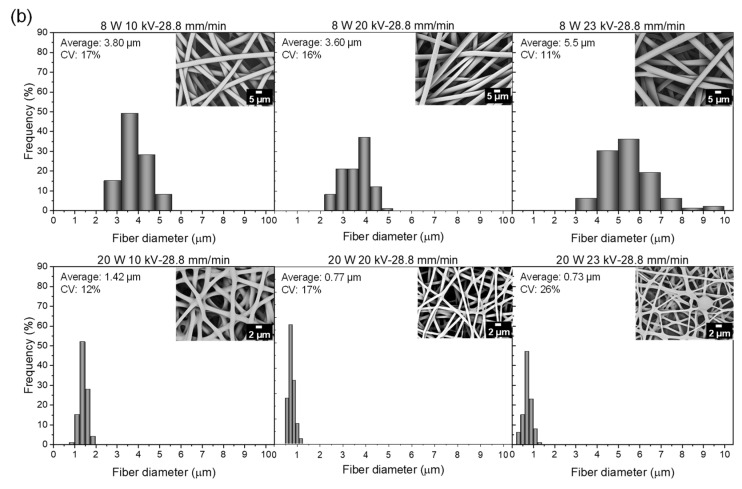
(**a**) Effect of feeding rate on fiber diameter distribution for the applied voltage of 20 kV and laser power of 8 and 20 W, and (**b**) effect of applied voltage on fiber diameter distribution for the feeding rate of 28.8 mm/min and laser power of 8 and 20 W. SEM images of corresponding fiber web are also shown.

**Figure 8:**
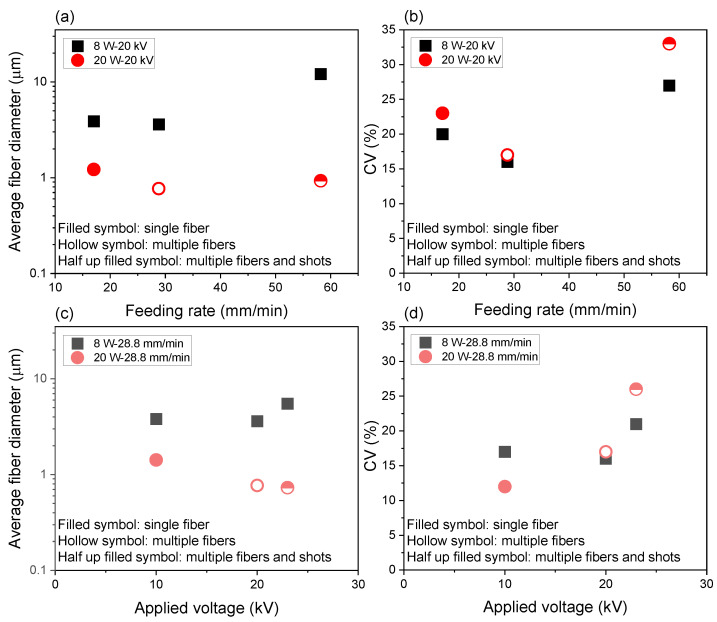
Variations of (**a**) average fiber diameter and (**b**) its coefficient of variation (CV) with change in feeding rate for the applied voltage of 20 kV and laser power of 8 and 20 W, and variations of (**c**) average fiber diameter and (**d**) its CV with change in the applied voltage for the feeding rate of 28.8 mm/min and laser power of (**a**) 8 W and (**b**) 20 W.

Diameter profiles of the spin-line in the LES of various spinning conditions analyzed from the captured images (Figure 3) are shown in Figure 6. In these figures, fiber diameters analysed from the SEM images of the prepared web samples, which are presented in Figure 7 and Figure 8, were also plotted at 60 mm from the nozzle (the nozzle tip to collector distance) for comparison. Similar to the fiber diameter profiles of PLLA in LES reported in our previous research [21], it was found that the fiber diameter profiles along spin-line (*x* axis) of PLCL showed the characteristics of initial increase followed by the steep reduction in fiber diameter to the order of 10 μm at the tip of the Taylor cone. The increase and decline in fiber diameter are ascribed to the cohesive force related to surface tension and repulsive electric force for fiber stretch, respectively. In our previous study, it was found that the up-stream movement of the swelling region and steeper fiber thinning led to the production of thinner fibers in the web [21].

In Figure 6a, a higher maximum fiber diameter along with the downstream movement of the swelling region was observed when the feeding rate was increased from 17.0 to 72.2 mm/min at the laser power of 8 W and applied voltage of 20 kV. Fiber diameter decreased more mildly downstream with the increase in the feeding rate. For the laser power of 20 W and applied voltage of 20 kV (Figure 6b), a noticeable increase in maximum fiber diameter and more intense reduction rate in fiber diameter was detected when the feeding rate was increased to 43.3 mm/min where multiple jets and shots started to be ejected from the swelling region.

Figure 6c,d illustrates the dependence of fiber diameter profiles on applied voltage from 6–23 kV at the laser power of 8 and 20 W under the constant feeding rate of 28.8 mm/min. For the laser power of both 8 and 20 W, the size of the swelling region did not change, while its position moved downstream with the increase in applied voltage. For the laser power of 8 W, the reduction in fiber diameter in the downstream proceeded gradually when the applied voltage was lower than 15 kV, while diameter decreased more steeply for the laser power of 20 W and multiple jets appeared when applied voltage exceeded 15 kV.

#### 3.1.4. Diameter of Fibers in the Web

Dependence diameter distribution of laser electrospun PLCL fibers in the web on feeding rate and applied voltage are shown in Figure 7a,b with insets of SEM images of fibers.

For the effect of feeding rate shown in Figure 7a, at the laser power of 8 W, prepared fibers were well-separated, and the fiber diameter and its distribution were fairly large, especially at the feeding rate of 58.2 mm/min. These results are inconsistent with the captured images of fiber thinning behavior shown in Figure 6. On the other hand, at the laser power of 20 W, the fusion and interconnection of fibers was observed for all feeding rates where the degree of fusion was enhanced, and shots were observed with the increase in the feeding rate.

For the effect of applied voltage shown in Figure 7b, fibers of fairly large diameter were well-separated at the laser power of 8 W, while the fusion of fibers was observed at 20 W, and shots were found at higher applied voltages where multiple fibers were formed. The fusion of fibers in the webs can be attributed to the insufficient cooling and/or low level of crystallization of PLCL fibers before reaching the collector in the spinning process.

The effects of feeding rate and applied voltage on the averaged fiber diameter and its coefficient of variation are summarized in Figure 8. The relation between the processing condition and fiber diameter was not straightforward, while it can be seen clearly that the formation of multiple jets resulted in the preparation of thinner fibers. In such conditions, however, there was a higher possibility for the emergence of shots in the spinning process and fusion of fibers in the resultant webs. Nevertheless, it was concluded that a combination of the laser power, applied voltage, and feeding rate of 20 W, 20 kV, and 28.8 mm/min was the optimum condition for the production of thinner fibers, where the average fiber diameter of 0.77 μm and its CV of 17% was achieved without the formation of shots.

### 3.2. Quantitative Estimation of Spinning Behavior

#### 3.2.1. Estimations of Fiber Velocity and Strain Rate

**Figure 9:**
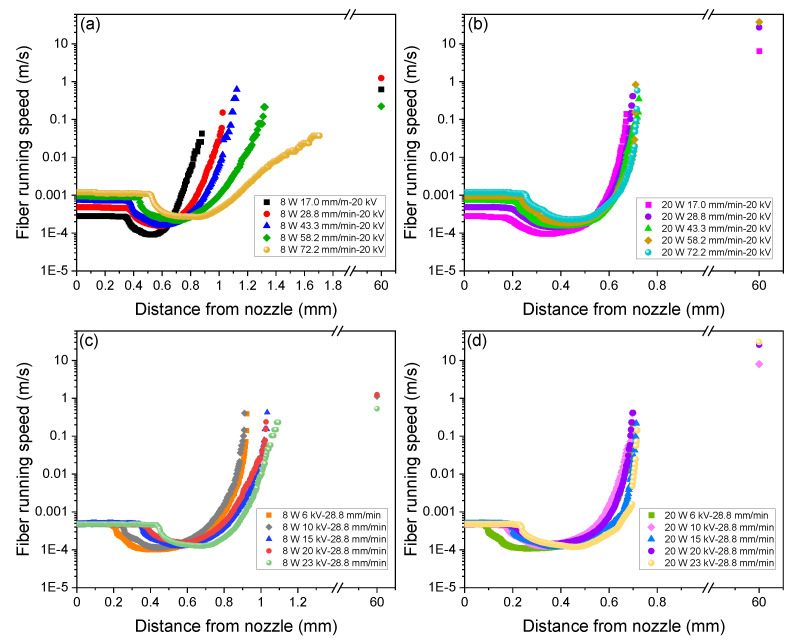
Effect of feeding rate on fiber running speed profiles for the applied voltage of 20 kV and laser power of (**a**) 8 W and (**b**) 20 W, and effect of applied voltage on fiber running speed profiles for the feeding rate of 28.8 mm/min and laser power of (**c**) 8 W and (**d**) 20 W.

**Figure 10:**
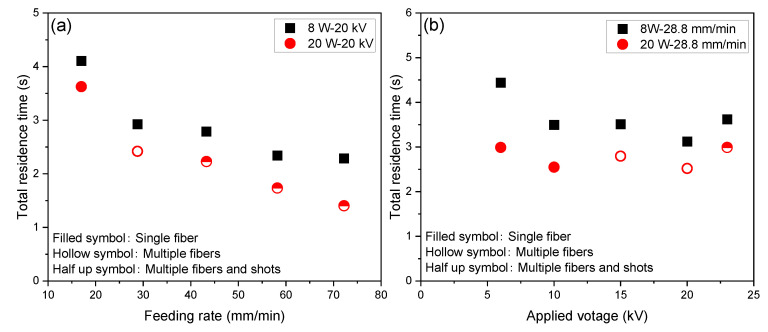
Variations of total residence time with changes in (**a**) feeding rate and (**b**) applied voltage at laser power of 8 and 20 W.

Based on the measurement of fiber diameter in the swelling and thinning regions, the running speed of fiber (spin-line velocity) at each position along the spin-line was calculated using Equation (2) as shown in Figure 9. Strain rate can be deduced by the derivation of fiber velocity based on Equation (3). Furthermore, residence time of the polymer in the spin-line was calculated through the integration of the reciprocal of velocity based on Equation (4). Variations of total residence time with feeding rate and applied voltage are shown in Figure 10a,b. Variations of strain rate and residence time with the distance from the nozzle for various spinning conditions are shown in Figures S1 and S2 (see Supplementary Materials).

Analysis on spin-line velocity revealed that the final fiber velocity estimated from the diameter of fibers in the web was at most approximately 6.3 m/s (=378 m/min). It should be noted that there was an overestimation of fiber velocity for the condition of the ejection of multiple jets and shots. The maximum fiber velocity from the on-line measurement was approximately 1 m/s (= 60 m/min). This limitation corresponds to the resolution of fiber diameter in the captured images.

For the laser power of 8 W, a steep increase in the strain rate shifted to downstream with the increase in the feeding rate, while at the laser power of 20 W, the strain rate increased steeply at around 0.6 mm for all feeding rates. These behaviors correspond to the diameter and velocity profiles described previously. It is important to note that the estimated maximum strain rate in the spin-line, which was strongly affected by the final diameter observed in the spin-line, reached at least a few thousand reciprocal seconds to ten thousand reciprocal seconds. These values are extremely high in comparison with the ordinary melt spinning conditions. Similar values were reported for the strain rate of the neck-like deformation in the high-speed spin-line [34]. Wang et.al. reported a lower strain rate for the solution electrospinning [35]. It was also found that the residence time of the polymer in the spin-line was within the range from 1.3 to 4.5 s. The relatively long residence time was due to the existence of the swelling region in the LES. Residence time was shorter for the conditions of a higher feeding rate, higher applied voltage, and higher laser power.

#### 3.2.2. Estimation of Tension and Stress Profiles

**Figure 11:**
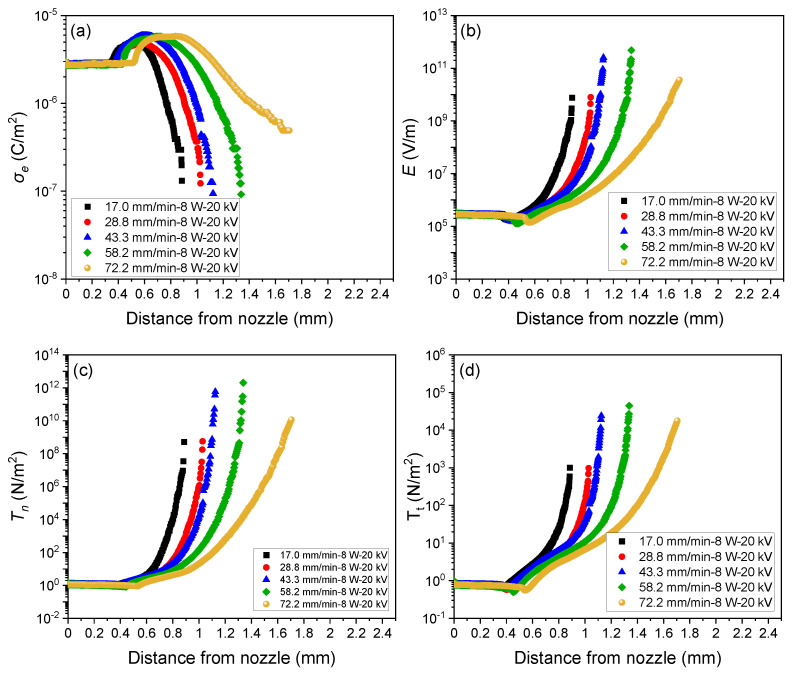

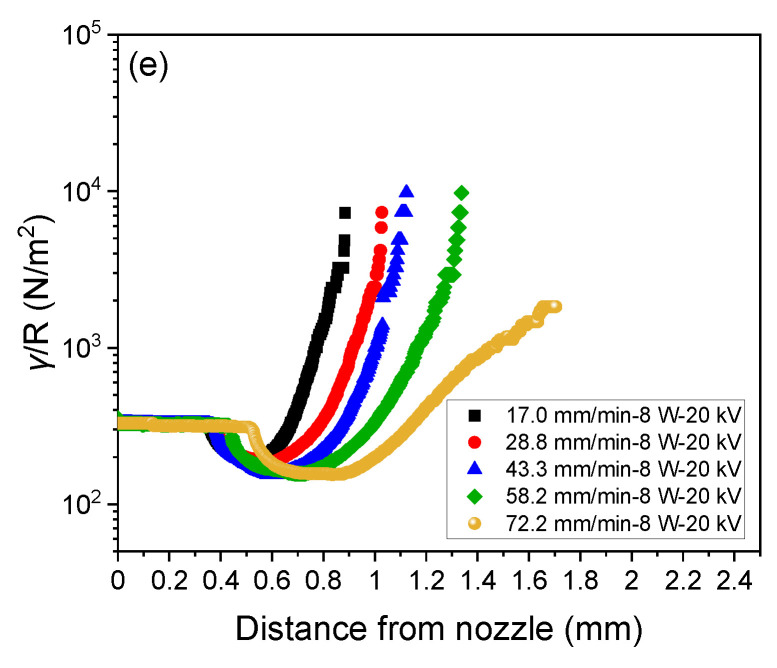
Effect of feeding rate on (**a**) charge density profiles, (**b**) electric field profiles, (**c**) normal stress profiles, (**d**) tangential stress profiles, and (**e**) cohesive force profiles for the applied voltage of 20 kV and laser power of 8 W.

The result of the analyses on the variations of surface charge density (*σe*), electric field (E), normal stress ($T_{n}$), and tangential stress ($T_{t}$) along the spin-line for different feeding rates at the laser power of 8 W and applied voltage of 20 kV are shown in Figure 11. The inward force (γ/R), which originated from the surface tension, is also plotted for comparison. Analyses were conducted based on the fiber diameter and fiber velocity distributions in the swelling region shown in Figure 6 and Figure 9. Similar results for the effects of feeding rate and applied voltage are shown in Figures S3–S5 (see Supplementary Materials).

The profiles of $\sigma_{e}$, E, $T_{n}$, $T_{t}$, and γ/R along the *x*-axis can be explained as follows. The surface charge density $\sigma_{e}$ firstly increased from the initial value of the order of 10^−6^ C/m^2^ with the expansion in fiber diameter and subsequently decreased with fiber thinning to the value of the order of 10^−7^ C/m^2^. Based on the slender body model, the steep fiber thinning caused the sharp increase in the electric field, which caused the acceleration of fiber thinning. Eventually, the strain rate reached a maximum value at the cone apex, i.e., near the tip of the swelling region. As in the case of the electric field, normal and tangential stresses, $T_{n}$ and $T_{t}$, also increased with fiber thinning and reached maximum values at the cone apex because normal and tangential stresses are dependent on the surface electron density and electric field. Variation in the electric field has a strong relationship with change in strain rate, that is, a higher electric field caused a higher strain rate. In addition, the stresses $T_{n}$ and $T_{n}$ are considered to prevent and promote the stabilization of jet ejection from the cone apex, respectively. On the other hand, the inward force caused by surface tension varied depending on the variation of fiber diameter along the spin-line with the assumption of constant surface tension.

The relation between these parameters on fiber formation behavior can be recognized by plotting the variations of tension imposed on the spin-line, as shown in Figure 12 as magnified views. The variations of tension and tensile stress imposed on the spin-line were also plotted in Figures S6–S13 (see Supplementary Materials).

Spin-line tension and stress were estimated based on Equations (14) and (15), respectively. In these figures, tension and stress related to the inertial force, surface tension force, force caused by electric charge and electric field, and air-friction force are plotted against the distance from the nozzle. Total tension and stress, which correspond to the rheological force and stress, are also plotted. It should be noted that initial tension was assumed to be zero and, therefore, only relative change in these parameters can be discussed.

From these figures, it can be recognized generally that the term related to the surface tension dominated the tension and stress in the upstream where the thickening of fiber diameter occurred. With the increase in the distance from the nozzle, and when the thinning of the swelling region started, surface tension tried to prevent the thinning while the electric force tried to extend the filament. Both effects were almost balanced at a certain point. Downstream, the effect of electric force increased steeply and dominated the spin-line tension, which contributed to the further thinning of the filament. Accordingly, tension and stress showed a maximum approximately at the distance from 0.6 to 1.0 mm. It was found that the maximum value decreased with the increases in the feeding rate and applied voltage. The variation of the maximum value was found to be more significant for stress than tension. It appears that the effect of air-friction was almost negligible, whereas the effect of inertial force became visible at the end part of the thinning behavior, where the reduction in fiber diameter and acceleration of fiber speed continued. Comparing this result with Figure 6 and Figure 9, it was noted that the lower peak value corresponded to the formation of thicker fibers for the condition of single-jet ejection. This cannot be applied when multiple jets were ejected under the conditions of high feeding rate, high applied voltage, and high laser power. To the best of our knowledge, this is the first attempt for the quantitative comparison in terms of the contribution of each force and stress components in the melt electrospinning process.

### 3.3. Analysis of the Structure and Properties of As-Spun Fibers and Webs

#### 3.3.1. DSC

**Figure 13:**
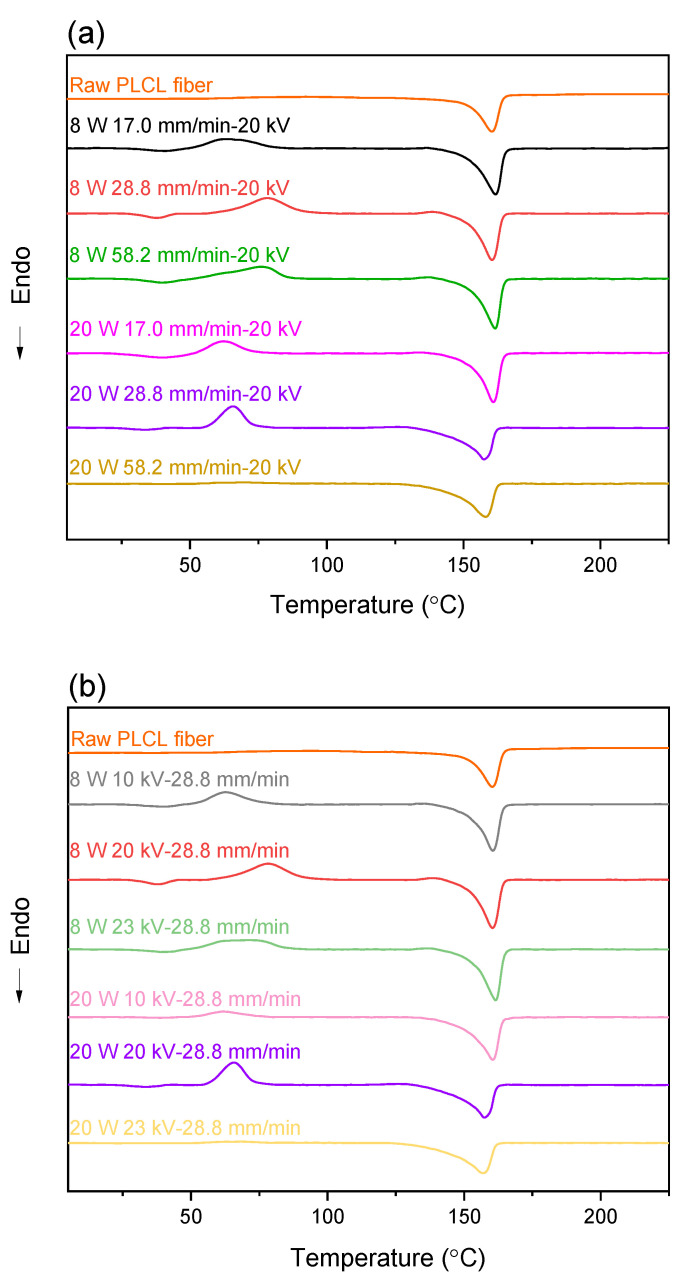
DSC thermograms of electrospun fibers obtained for conditions with various (**a**) feeding rate and (**b**) applied voltage at laser power of 8 and 20 W. Data of the raw PLCL fiber is also included.

DSC thermograms of raw PLCL fibers and electrospun fibers of various spinning conditions are shown in Figure 13. Glass transition, exothermic peak of cold crystallization, and endothermic peak of melting were observed consecutively with the increase in temperature, as summarized in Table 3. Glass transition temperature *T_g_* of PLCL fibers was near room temperature of approximately between 25 and 30 °C, which was about 30 degrees lower than the *T_g_* of pure PLLA. Reduction in *T_g_* is attributable to the copolymerization of ε-caprolactone. On the other hand, the single melting peak with the peak temperature of approximately 160 °C indicated that only the PLLA component crystallized in the PLCL used in this research [28].

The raw fiber did not exhibit a cold crystallization peak, indicating that the fiber was highly crystallized. On the other hand, most of the electrospun fibers showed a cold crystallization peak, and its peak temperature varied depending on the spinning conditions. In general, lowering of the cold crystallization temperature corresponds to the increase in the crystallization rate caused by the increase in the degree of molecular orientation. As shown in Figure 13a, the cold crystallization peak temperature increased with the increase in the feeding rate, while the fibers prepared at the laser power of 8 W exhibited a higher cold crystallization temperature in comparison with 20 W fibers. With the combination of the high feeding rate of 58.2 mm/min, high laser power of 20 W, and high applied voltage of 20 kV, however, the fiber did not show a cold crystallization peak. The disappearance of cold crystallization indicated the development of a crystalline structure in the process of LES. This is attributed to the existence of a large number of shots in the webs. A large diameter and low specific surface area of shots resulted in the lower cooling rate so that crystallization could be completed during the process.

Regarding the effect of applied voltage, Figure 13b indicates the increase in cold crystallization temperature with the increase in voltage for the laser power of both 8 and 20 W. Again, with the combination of high feeding rate, high laser power, and high applied voltage, the fiber did not show a cold crystallization peak. It was also recognized that the spinning condition with the combination of high feeding rate, high laser power, and high applied voltage resulted in the ejection of multiple jets and shots during the spinning process, which may lead to the appearance of a lower melting peak temperature of prepared fibers.

#### 3.3.2. Birefringence Analysis

Molecular orientation developed during electrospinning is considered to be a key factor determining the properties of electrospun fibers. In this study, molecular orientation was evaluated through birefringence measurement conducted using a polarizing microscope.

**Figure 14:**
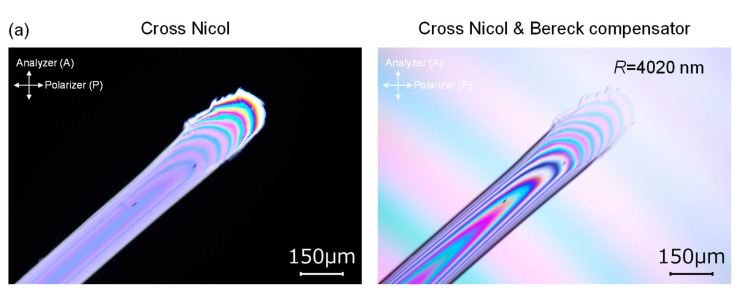

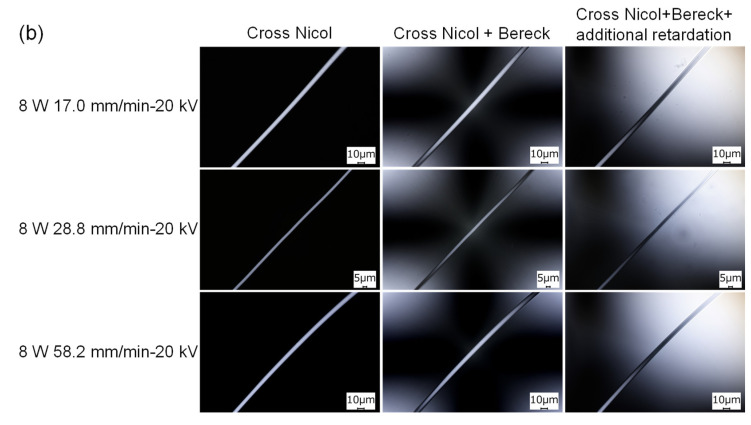
Micrographs of the fibers observed under a polarizing microscope: (**a**) raw fiber for LES and (**b**) electrospun fibers obtained for conditions with various feeding rate at laser power of 8 W. Cross Nicol, Cross Nicol + Bereck, and Cross Nicol + Bereck + additional retardation correspond to under cross-polarization condition, cross-polarization condition using the Bereck compensator without optical retardation, and using the Bereck compensator with optical retardation, respectively.

Micrographs of the raw fiber with an oblique cross-section captured under a polarizing microscope are shown in Figure 14a. Observation was conducted under the crossed polarization (Cross Nicol) without or with the insertion of a Bereck compensator. Position of the black interference fringe shifted near to the fiber surface with the addition of the optical retardation of 4020 nm using the compensator. By dividing the estimated optical retardation by the fiber diameter (= optical path length), birefringence was evaluated.

Images of the electrospun fibers observed under the polarizing microscope for the effect of feeding rate at the laser power of 8 W are shown in Figure 14b. Similar images for the effect of applied voltage at the laser power of 8 W are shown in Figure S14 (see Supplementary Materials). For the fibers prepared under various spinning conditions, observation was conducted under the crossed polarization without the compensator (Cross Nicol), with the insertion of the compensator with the neutral rotation angle of calcite crystal (Cross Nicol + Bereck), and with the insertion of the Bereck compensator with the tilting of calcite crystal to add optical retardation (Cross Nicol + Bereck + additional retardation). Through adjusting the position of the black interference fringe, as shown in the figure, optical retardation was estimated, and birefringence of the fibers were calculated by dividing the retardation with the fiber diameter.

**Figure 15:**
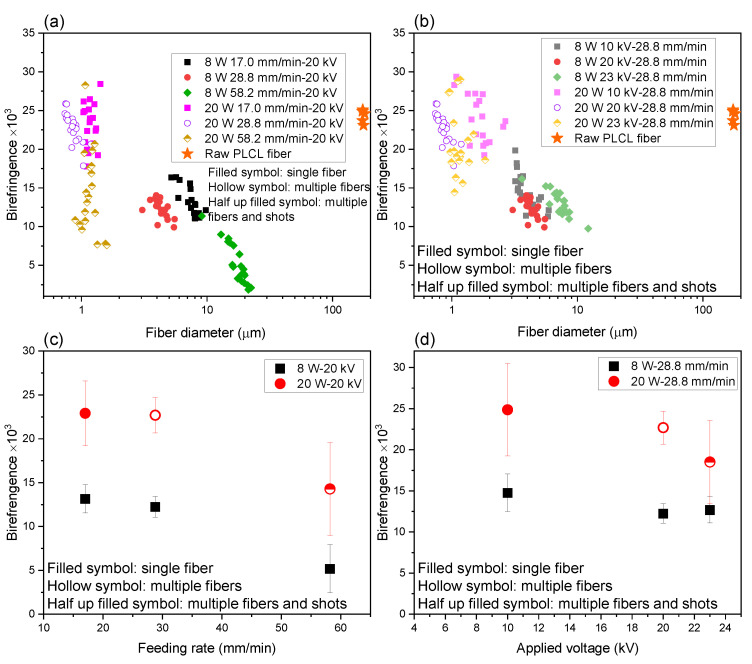
Correlation between fiber diameter and birefringence of electrospun fibers obtained for conditions with various (**a**) feeding rate and (**b**) applied voltage at laser power of 8 and 20 W. Data of the raw PLCL fiber is also included. Variations of averaged birefringence and its distribution with changes in (**c**) feeding rate and (**d**) applied voltage at laser power of 8 and 20 W.

The relationship between birefringence and fiber diameter for the raw fiber and electrospun fibers obtained at various LES conditions of feeding rate and applied voltage are plotted in Figure 15a,b, respectively. The raw fiber exhibited relatively high birefringence of approximately 25 × 10^−3^. For the electrospun fibers, at the laser power of 8 W, birefringence increased with the reduction in fiber diameter, whereas at the laser power of 20 W, fibers with the diameter of approximately 1 μm but having a wide range of birefringence were obtained. At the laser power of 20 W and under the conditions of high applied voltage and high feed rate, where multiple jets and shots were observed, it was found that the fibers with bimodal distribution of high and low birefringence were obtained.

The effects of feed rate and applied voltage on averaged birefringence and its distribution are summarized in Figure 15c,d. It is obvious that the fibers with higher birefringence were produced by applying the laser power of 20 W rather than 8 W. It was also found that the birefringence tended to decrease with the increase in feeding rate and applied voltage, especially for the laser power of 20 W.

The increase in molecular orientation with the reduction in fiber diameter was also found for the analyses of electropsun PLLA and PCL fibers and PE fibers [36,37].

#### 3.3.3. WAXD Analysis

**Figure 16:**
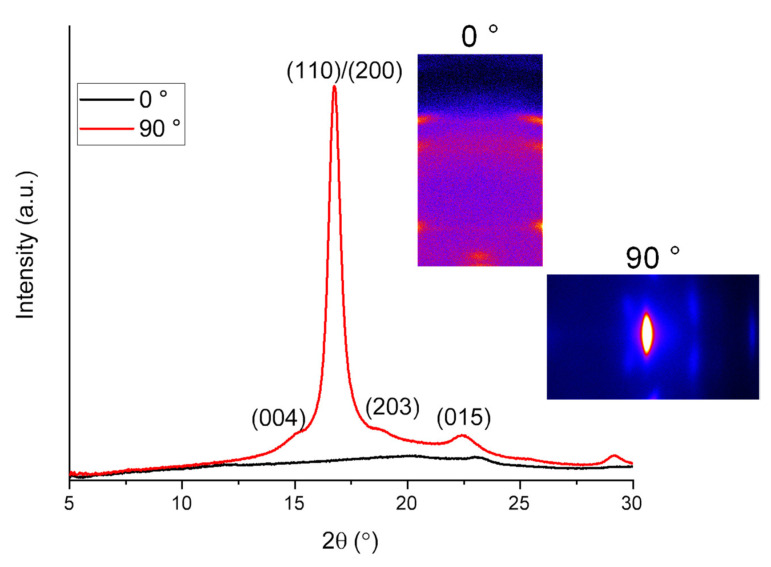
Wide-angle X-ray diffraction (WAXD) profiles and two-dimensional (2D) patterns at azimuthal angles of 0 and 90° for a bundle of the raw fiber.

The WAXD intensity profiles of raw fiber at azimuthal angles of 0° (meridian) and 90° (equator) are shown in Figure 16, along with the insertion of two-dimensional (2D) intensity patterns near the equator and meridian. Distinct peaks in the WAXD intensity profile acquired at 0° and 90° suggested the presence of an anisotropic crystalline structure in the raw fiber, and crystalline reflections in the 2D pattern implied the orientation of crystalline c-axis along the fiber axis. The crystalline peaks observed at 2θ = 15.0°, 16.7°, 19.1°, and 22.4° can be assigned to the (004), (110)/(200), (203), and (015) reflections of the α-form crystal of PLLA with the pseudo-orthorhombic unit cell of a = 1.07 nm, b = 0.595 nm, and c = 2.78 nm, which contains two 10_3_ helices [38]. It should be noted that the crystalline peak of PCL was not observed because the composition of PCL in the PLCL copolymer was too low to form PCL crystals.

**Figure 17:**
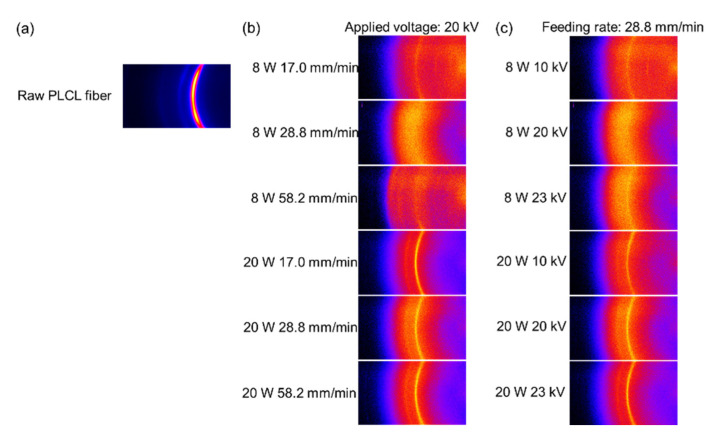
WAXD 2D patterns of (**a**) powder-like raw fiber and electrospun fiber webs obtained for conditions with various (**b**) feeding rate and (**c**) applied voltage at laser power of 8 and 20 W.

**Figure 18:**
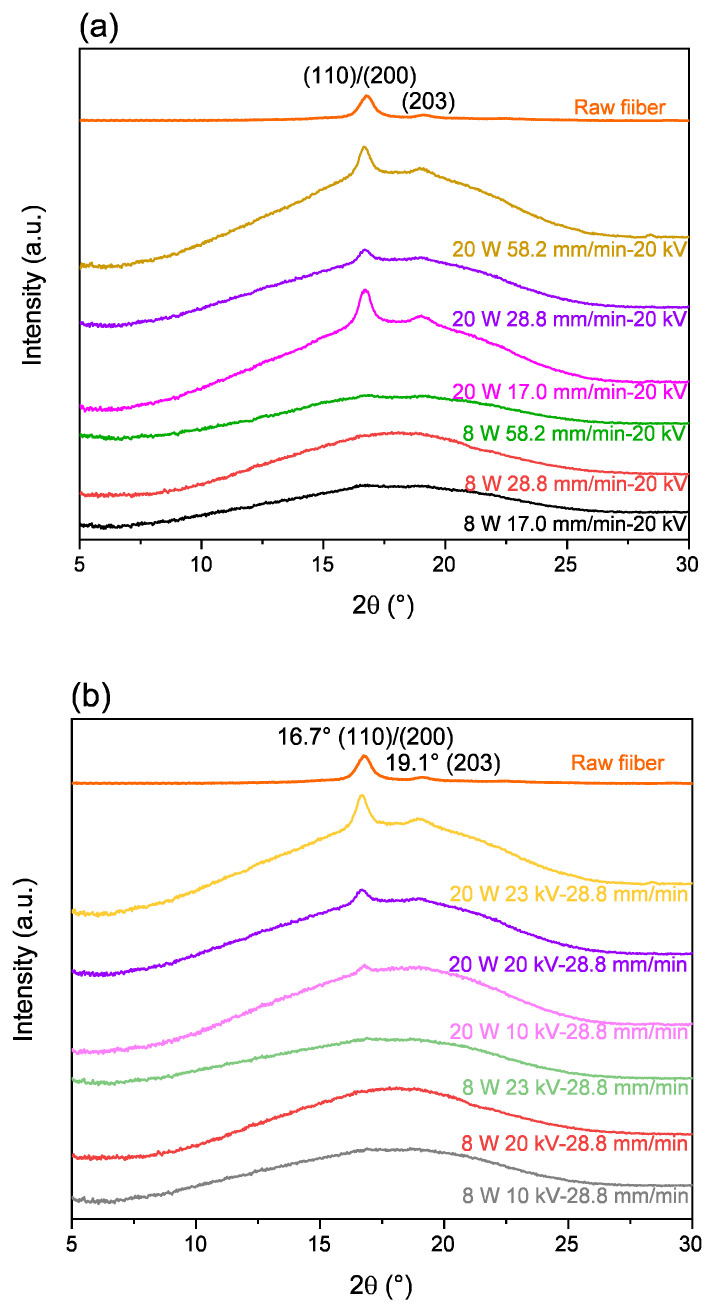
WAXD profiles of electrospun fiber webs obtained for conditions with various (**a**) feeding rate and (**b**) applied voltage at laser power of 8 and 20 W. Data of the raw PLCL fiber is also included.

WAXD 2D patterns for the electrospun webs of various processing conditions are shown in Figure 17. WAXD intensity profiles obtained from these patterns are shown in Figure 18. In this figure, the intensity distribution of randomly oriented chopped raw fibers is also shown for comparison. At the laser power of 8 W, a broad amorphous halo was observed with a slight indication of the existence of a small amount of crystalline structure at each feed rate and applied voltage condition. On the other hand, at the laser power of 20 W, distinct crystalline peaks were observed at 16.7° and 19.1° for all feed rates and applied voltage conditions. It was obvious that the crystallization of the electrospun fibers was promoted by the increase in the laser power for heating.

#### 3.3.4. Orientation and Crystallization of Electrospun Fibers

**Figure 19:**
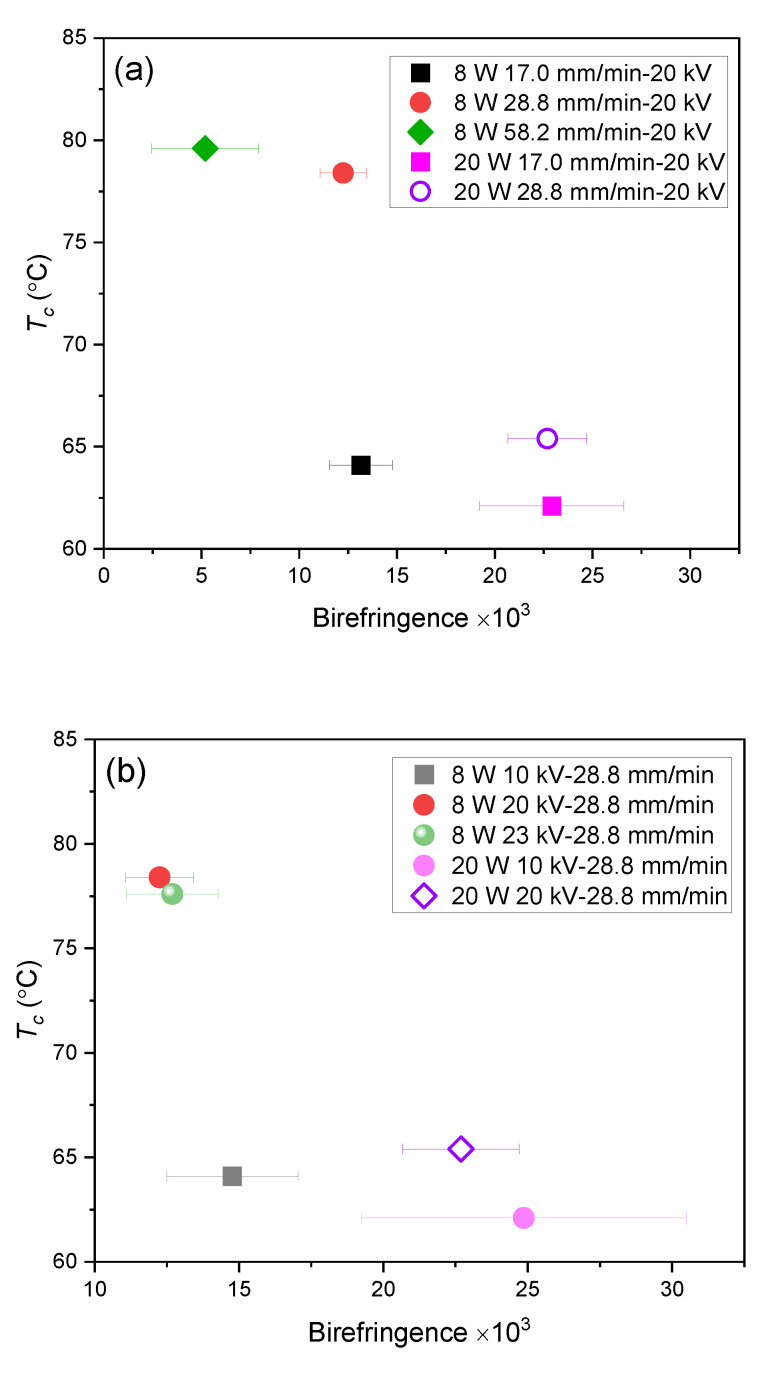
Correlation between *T_c_* and birefringence for conditions with various (**a**) feeding rate and (**b**) applied voltage at laser power of 8 and 20 W.

The relation between structural parameters obtained through the measurements of DSC, birefringence, and WAXD can be summarized as follows. In general, the cold crystallization temperature decreased with the increase in birefringence, as shown in Figure 19. This is due to the acceleration of the crystallization rate caused by the molecular orientation. From the WAXD analysis, fibers prepared with the laser power of 8 W only presented as an amorphous halo, whereas the fibers prepared with the laser power of 20 W exhibited crystalline reflections. On the other hand, crystallinity of the 8 W fibers evaluated from the result of DSC measurement was in the range from 16–20%. It should be noted that even for the amorphous fibers, the DSC analysis generally gives a certain value of crystallinity if the fibers have a certain degree of molecular orientation [21].

It was also found that for the fibers prepared with the multiple ejection of jets and shots in the LES process, i.e., the condition with high laser power, high feeding rate, and high applied voltage, cold crystallization disappeared, and the melting temperature became lower in the DSC analysis. The fusion of fibers in the web occurred in these conditions, and birefringence of the fibers exhibited bimodal distribution. These results suggested that the crystallization proceeded in the spinning process even for the fibers with low birefringence and shots. This peculiar behavior is attributable to the effect of the history of elongational flow in the spinning process [39,40].

## 4. Conclusions

In this study, the dependence of fiber formation and properties on processing under various feeding rates and applied voltage was investigated.

Based on the in situ observation of fiber thinning behavior, three types of fiber thinning behavior in the swelling area including Type I: single jet, Type II: multiple jets, and Type III: multiple jets and shots was found. The higher laser power, feeding rate, and applied voltage were prone to produce multiple jets and shots, which resulted in thinner fibers. The thinnest fiber had a diameter of 0.77 μm with a coefficient of variation of 17% at a laser power of 20 W, a feeding rate of 28.8 mm/min, and applied voltage of 20 kV.

The mechanism of fiber formation was further discussed by the tension and stress along the spin-line corresponding to the inertial force, surface tension force, force caused by electric charge and electric field, and air-friction force. It was revealed that the dominant force is surface tension in the up-stream but electric force in the down-stream for fiber thinning. The maximum values of tension and stress appearing near the apex of the swelling region decreased with the increase in the feeding rate and voltage, which resulted in the thicker fiber in the case of single fiber ejection; however, it was not applicable in the case of the ejection of multiple fibers and shots generated under the conditions of high feeding speed, high applied voltage, and high laser power. The tensile and stress behaviors were almost unaffected by the air-friction but were affected by the inertial force at the end of the thinning process, where the reduction of fiber diameter and acceleration of fiber speed proceeded.

In addition, the properties of as-spun fibers prepared by LES were evaluated by the analyzing of molecular orientation and crystallization. From the WAXD analysis, fibers prepared with the laser power of 8 W presented as amorphous halo, whereas the fibers prepared with the laser power of 20 W exhibited crystalline reflections. The birefringence of the obtained as-spun fibers was higher at 20 W as compared to that at 8 W. Especially, for the high laser power of 20 W, the condition with high feeding rate and high applied voltage, where the fibers prepared with the multiple ejection of jets and shots in the LES process, showed the lower average value and the bimodal distribution of birefringence. In addition, for these conditions, the cold crystallization temperature disappeared, and the melting peak shifted to a lower temperature in the DSC thermograms. These results suggested that the crystallization was enhanced in the spinning process even for the fibers with shots and low birefringence.

## Figures and Tables

**Figure 12:**
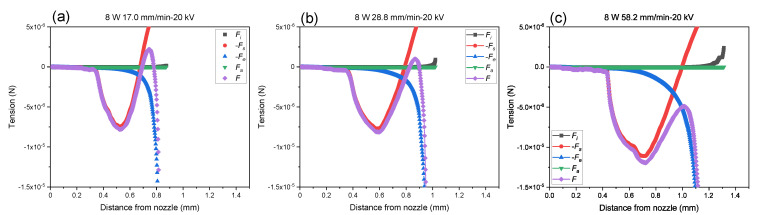
Magnified views of tension profiles for conditions with the feeding rate of (**a**) 17.0, (**b**) 28.8, and (**c**) 58.2 mm/min, applied voltage of 20 kV, and laser power of 8 W.

**Table 1:** Experimental conditions for LES process.

Laser-Nozzle Distance (mm)	Nozzle-Collector Distance (mm)	Laser Power (W)	Feeding Rate (mm/min)	Applied Voltage (kV)
ca. 0.8	60	8, 20	28.8	6
				10
				15
				20
				23
			17.0	20
			43.3	
			58.2	
			72.2	

**Table 2:** Parameters for processing conditions and material characteristics used for theoretical analysis.

Parameters	Unit	Values	Source
Applied voltage, *V*	V	10 × 10^3^, 20 × 10^3^, 23 × 10^3^	Experimental condition
Distance between nozzle and collector, *L_fin_*	m	60 × 10^−3^	Experimental condition
Applied external electric field, *E_∞_*(*x*)	V/m	17 × 10^4^, 33 × 10^4^, 38 × 10^4^	Experimental condition
Dielectric constant of PLCL, *ε*	F/m	2.655 × 10^−11^	Measurement
Small section along the fiber running direction, *dx*	m	2.2 × 10^−6^	Measurement
Radius of jet (fiber), *R(x)*	m	-	Measurement
Velocity of jet (fiber), *v(x)*	m/s	-	Measurement
Electric field, *E(x)*	V/m	-	Measurement and estimation
Surface charge density of jet (fiber), *σ_e_* (*x*)	C/m^2^	-	Measurement and estimation
Total current of jet (fiber), *I*	A	-	Measurement and estimation by Equations (5) and (6)
Dielectric constant of the ambient air, *ε*_0_	F/m	8.854 × 10^−12^	Literature [8]
Surface tension of PLCL, *γ*	N/m	3.25 × 10^−2^	Literature [9]
Conductivity of PLCL, *K*	S/m	1 × 10^−9^	Literature [10]
Fiber density, *ρ*	kg/m^3^	1211	Estimation

**Table 3:** DSC results for raw PLCL fiber and electrospun fibers obtained for various conditions.

LES Conditions	*T_g_* (°C)	*T_c_* (°C)	*T_m_* (°C)	*X_c_* (%)
Raw PLCL fiber	33.2	-	162.9	37.7
8 W 17.0 mm/min-20 kV	33.4	64.1	161.4	19.8
8 W 28.8 mm/min-20 kV	32.3	78.4	160.6	16.1
8 W 58.2 mm/min-20 kV	32.5	79.6	160.9	16.8
20 W 17.0 mm/min-20 kV	30.7	62.1	160.7	26.3
20 W 28.8 mm/min-20 kV	26.8	65.4	157.4	19.2
20 W 58.2 mm/min-20 kV	25.7	-	157.7	33.1
8 W 10 kV-28.8 mm/min	31.7	62.6	160.5	18.6
8 W 20 kV-28.8 mm/min	32.3	78.4	160.6	16.1
8 W 23 kV-28.8 mm/min	33.1	72.1	161.6	18.8
20 W 10 kV-28.8 mm/min	24.9	62.8	160.5	17.9
20 W 20 kV-28.8 mm/min	26.8	65.4	157.0	19.2
20 W 23 kV-28.8 mm/min	32.9	-	157.4	33.3

## Data Availability

Not applicable.

## Supplementary Materials

The following are available online at https://www.mdpi.com/article/10.3390/polym14122511/s1, Figure S1: Effect of (a) feeding rate on strain rate profiles for the applied voltage of 20 kV and laser power of 8 W and 20 W, and effect of (b) applied voltage on strain rate profiles for the feeding rate of 28.8 mm/min and laser power of 8 W and 20 W. Figure S2: Effect of feeding rate on residence time profiles for the applied voltage of 20 kV and laser power of (a) 8 W and (b) 20 W, and effect of applied voltage on residence time profiles for the feeding rate of 28.8 mm/min and laser power of (c) 8 W and (d) 20 W. Figure S3: Effect of feeding rate on (a) charge density profiles, (b) electric field profiles, (c) normal stress profiles, (d) tangential stress profiles, and (e) cohesive force profiles for the applied voltage of 20 kV and laser power of 20 W. Figure S4: Effect of applied voltage on (a) charge density profiles, (b) electric field profiles, (c) normal stress profiles, (d) tangential stress profiles, and (e) cohesive force profiles for the feeding rate of 28.8 mm/min and laser power of 8 W. Figure S5: Effect of applied voltage on (a) charge density profiles, (b) electric field profiles, (c) normal stress profiles, (d) tangential stress profiles, and (e) cohesive force profiles for the feeding rate of 28.8 mm/min and laser power of 20 W. Figure S6: Tension profiles for conditions with the feeding rate of (a) 17.0, (b) 28.8, and (c) 58.2 mm/min, applied voltage of 20 kV and laser power of 8 W. Figure S7: Stress profiles for conditions with the feeding rate of (a) 17.0, (b) 28.8, and (c) 58.2 mm/min, applied voltage of 20 kV and laser power of 8 W. Figure S8: Tension profiles for conditions with the feeding rate of (a) 17.0, (b) 28.8, and (c) 58.2 mm/min, applied voltage of 20 kV and laser power of 20 W. Figure S9: Stress profiles for conditions with the feeding rate of (a) 17.0, (b) 28.8, and (c) 58.2 mm/min, applied voltage of 20 kV and laser power of 20 W. Figure S10: Tension profiles for conditions with the applied voltage of (a) 10, (b) 20, and (c) 23 kV, feeding rate of 28.8 mm/min and laser power of 8 W. Figure S11: Stress profiles for conditions with the applied voltage of (a) 10, (b) 20, and (c) 23 kV, feeding rate of 28.8 mm/min and laser power of 8 W. Figure S12: Tension profiles for conditions with the applied voltage of (a) 10, (b) 20, and (c) 23 kV, feeding rate of 28.8 mm/min and laser power of 20 W. Figure S13: Stress profiles for conditions with the applied voltage of (a) 10, (b) 20, and (c) 23 kV, feeding rate of 28.8 mm/min and laser power of 20 W. Figure S14: Micrographs of the fibers observed under a polarizing microscope: electrospun fibers for conditions with various applied voltage at laser power of 8 W. Cross Nicol, Cross Nicol + Bereck, and Cross Nicol + Bereck + additional retardation correspond to under cross-polarization condition, cross-polarization condition using the Bereck compensator without optical retardation, and using the Bereck compensator with optical retardation, respectively.
