# Supplementary material for: Laser-Assisted Melt Electrospinning of Poly(L-lactide-co-ε-caprolactone): Analyses on Processing Behavior and Characteristics of Prepared Fibers
Source: Polymers (Basel). 2022 Jun 20;14(12):2511. doi: 10.3390/polym14122511 (PMC9227632; doi:10.3390/polym14122511)
Supplement: Supplementary file 1 [file polymers-14-02511-s001.zip › polymers-1777269-supplementary.pdf]

polymers

*Article*

# Supplementary Materials:

# Laser-Assisted Melt Electrospinning of Poly(L-lactide-co-ε-caprolactone): Analyses on Processing Behavior and Characteristics of Prepared Fibers

**Zongzi Hou [1], Haruki Kobayashi [2], Katsufumi Tanaka [2], Wataru Takarada [3], Takeshi Kikutani [4,5], and Midori Takasaki [2,*]**

1 Doctoral Program of Materials Chemistry, Graduate School of Science and Technology, Kyoto Institute of Technology, Matsugasaki, Sakyo-ku, Kyoto 606-8585, Japan; d9871502@edu.kit.ac.jp
2 Faculty of Materials Science and Engineering, Matsugasaki, Sakyo-ku, Kyoto Institute of Technology, Kyoto 606-8585, Japan; haruki@kit.ac.jp (H.K.); ktanaka@kit.ac.jp (K.T.)
3 Department of Materials Science and Engineering, Tokyo Institute of Technology, 2-12-1 Ookayama, Meguro-ku, Tokyo 152-8550, Japan; takarada.w.aa@m.titech.ac.jp
4 School of Materials and Chemical Technology, Tokyo Institute of Technology, 4259, Nagatsuta-cho, Midori-ku, Yokohama, Kanagawa 226-8503, Japan; kikutani.t.aa@m.titech.ac.jp
5 Center for Fiber and Textile Science, Kyoto Institute of Technology, Matsugasaki, Sakyo-ku, Kyoto 606-8585, Japan
* Correspondence: mitakas@kit.ac.jp

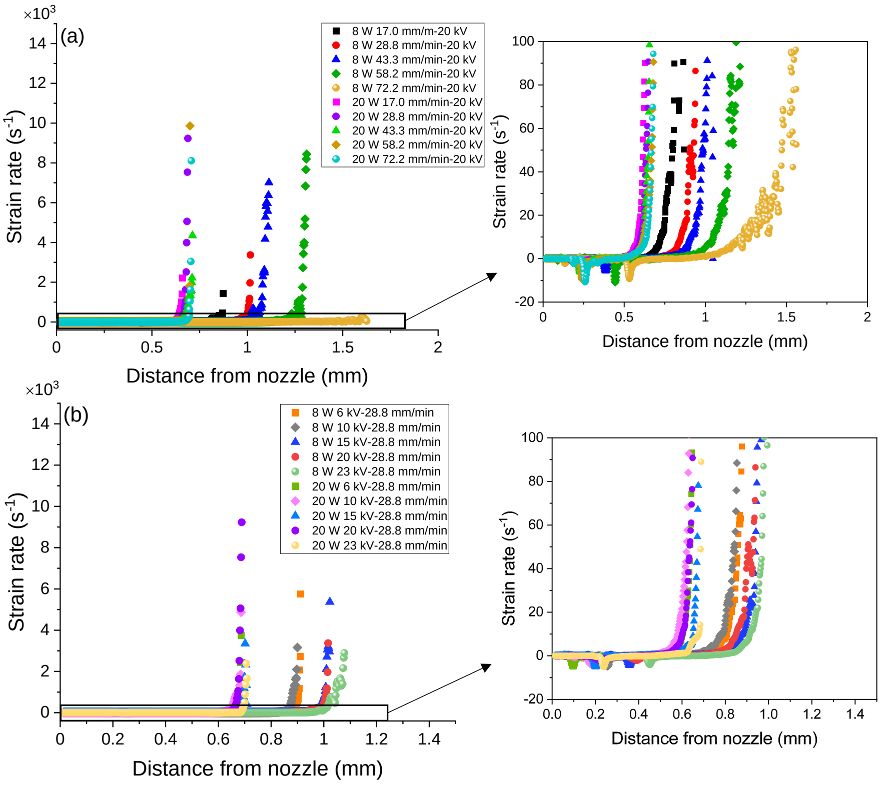


**Figure S1.** Effect of (a) feeding rate on strain rate profiles for the applied voltage of 20 kV and laser power of 8 W and 20 W, and effect of (b) applied voltage on strain rate profiles for the feeding rate of 28.8 mm/min and laser power of 8 W and 20 W.

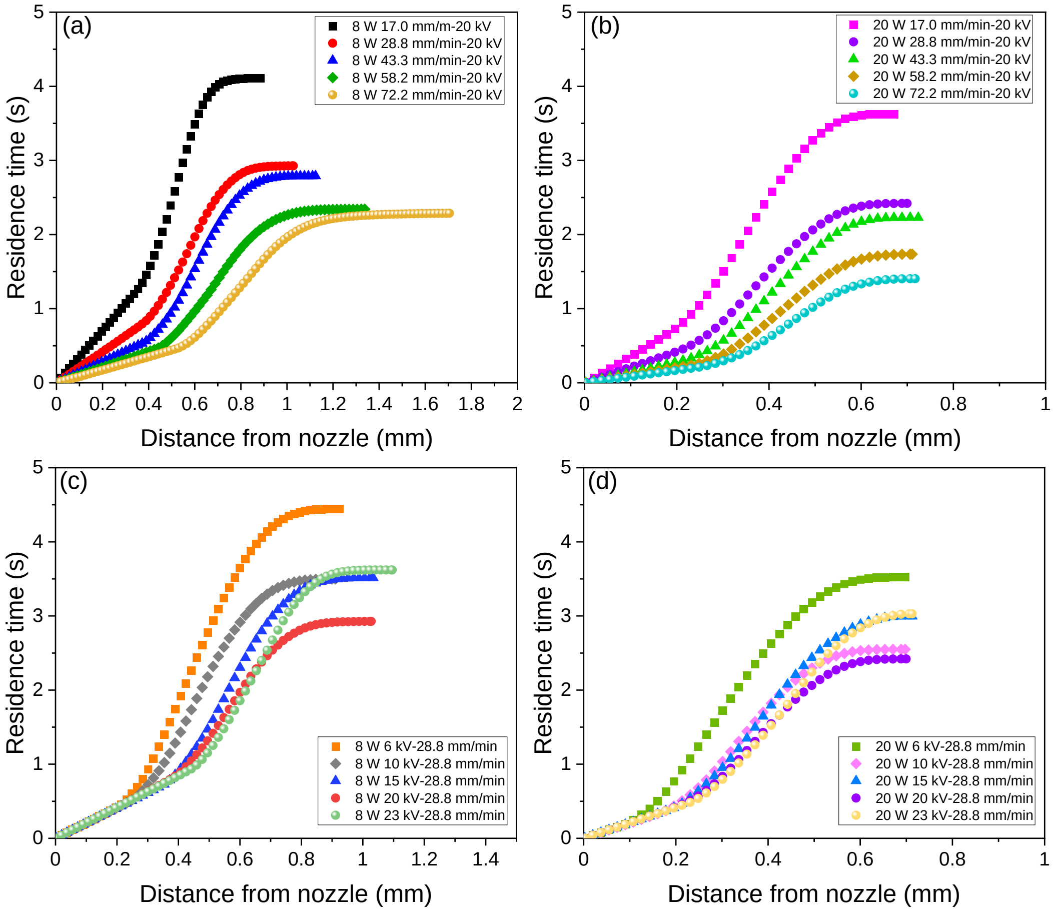


**Figure S2.** Effect of feeding rate on residence time profiles for the applied voltage of 20 kV and laser power of (a) 8 W and (b) 20 W, and effect of applied voltage on residence time profiles for the feeding rate of 28.8 mm/min and laser power of (c) 8 W and (d) 20 W.

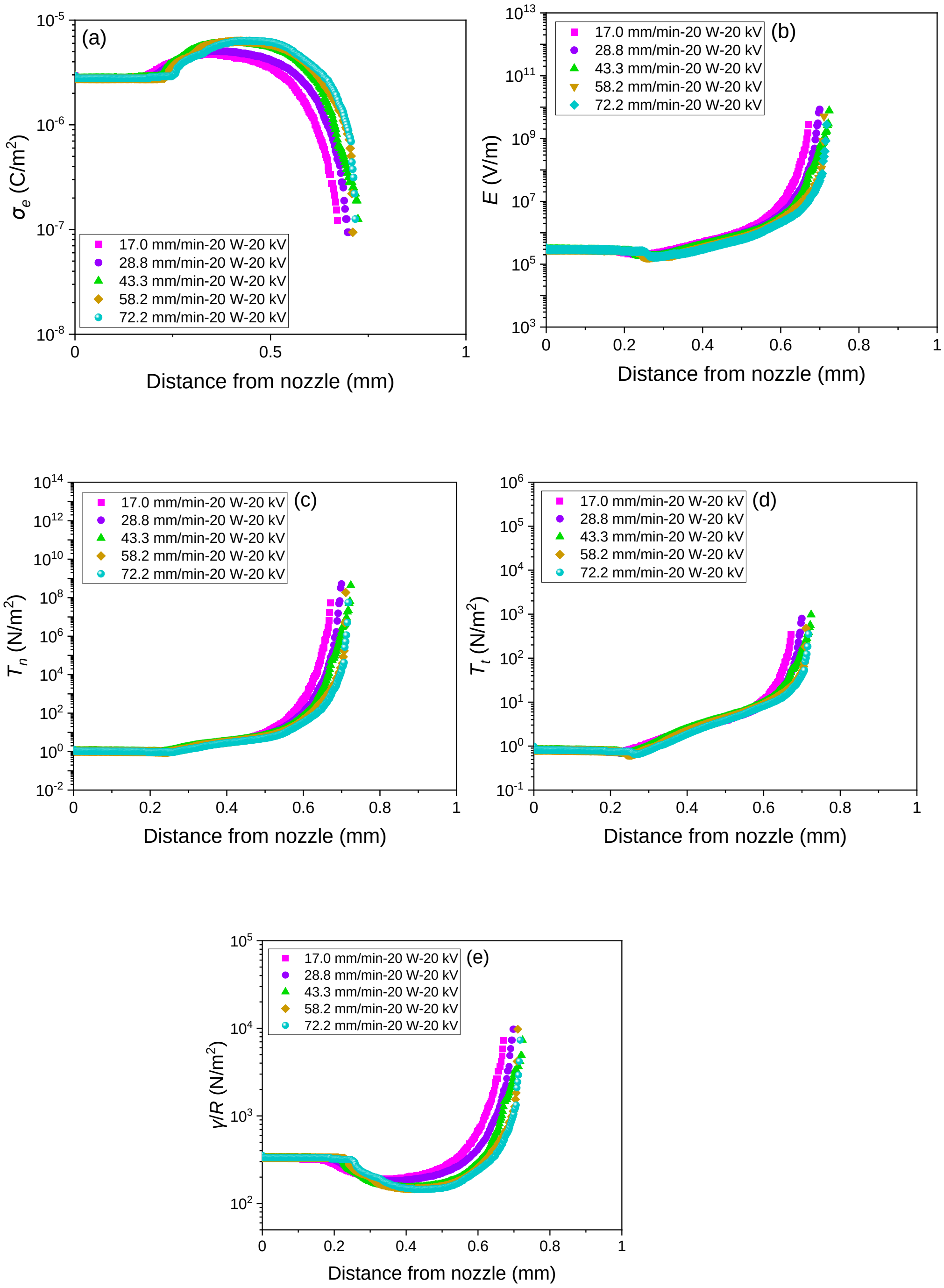


**Figure S3.** Effect of feeding rate on (a) charge density profiles, (b) electric field profiles, (c) normal stress profiles, (d) tangential stress profiles, and (e) cohesive force profiles for the applied voltage of 20 kV and laser power of 20 W.

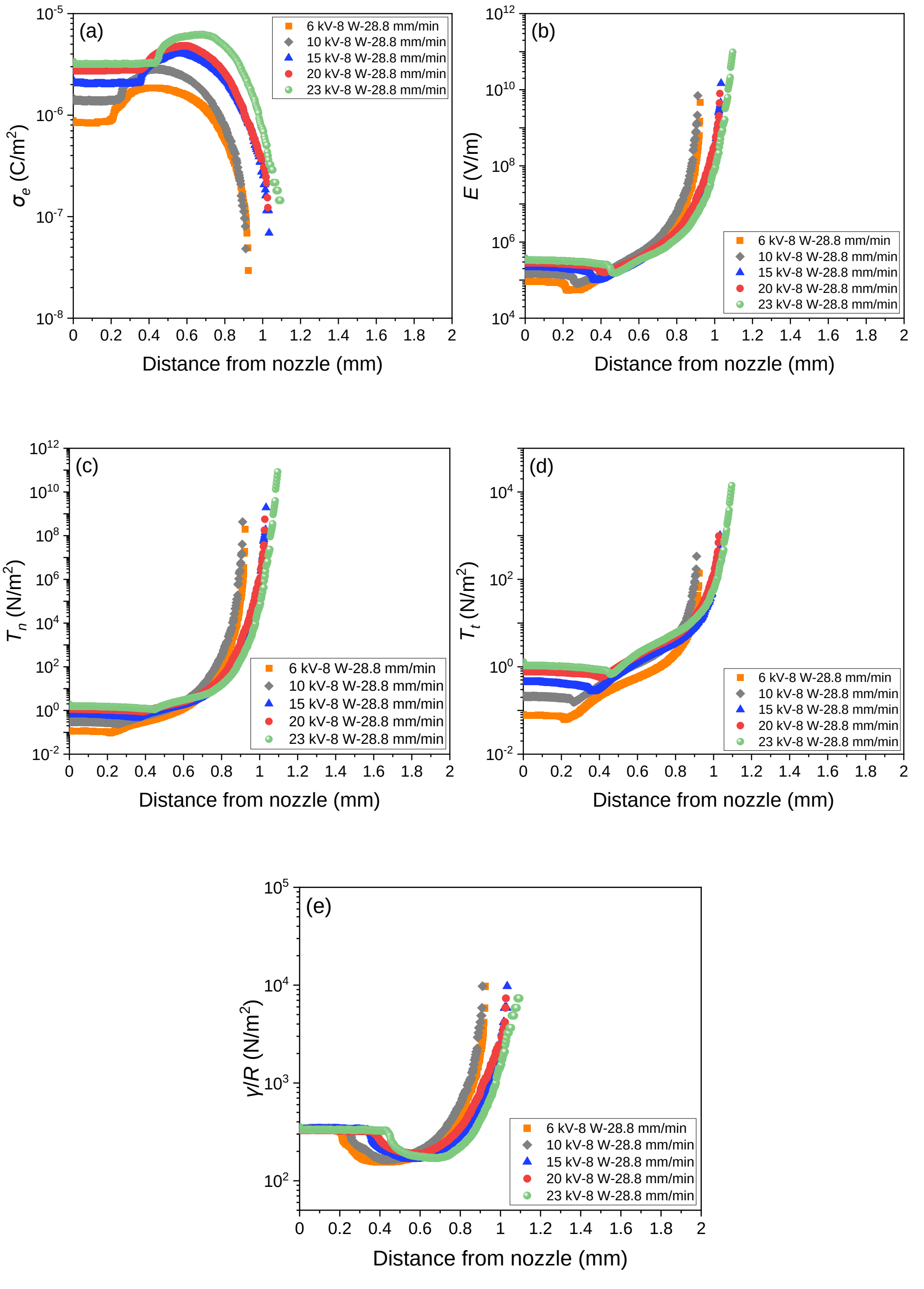


**Figure S4.** Effect of applied voltage on (a) charge density profiles, (b) electric field profiles, (c) normal stress profiles, (d) tangential stress profiles, and (e) cohesive force profiles for the feeding rate of 28.8 mm/min and laser power of 8 W.

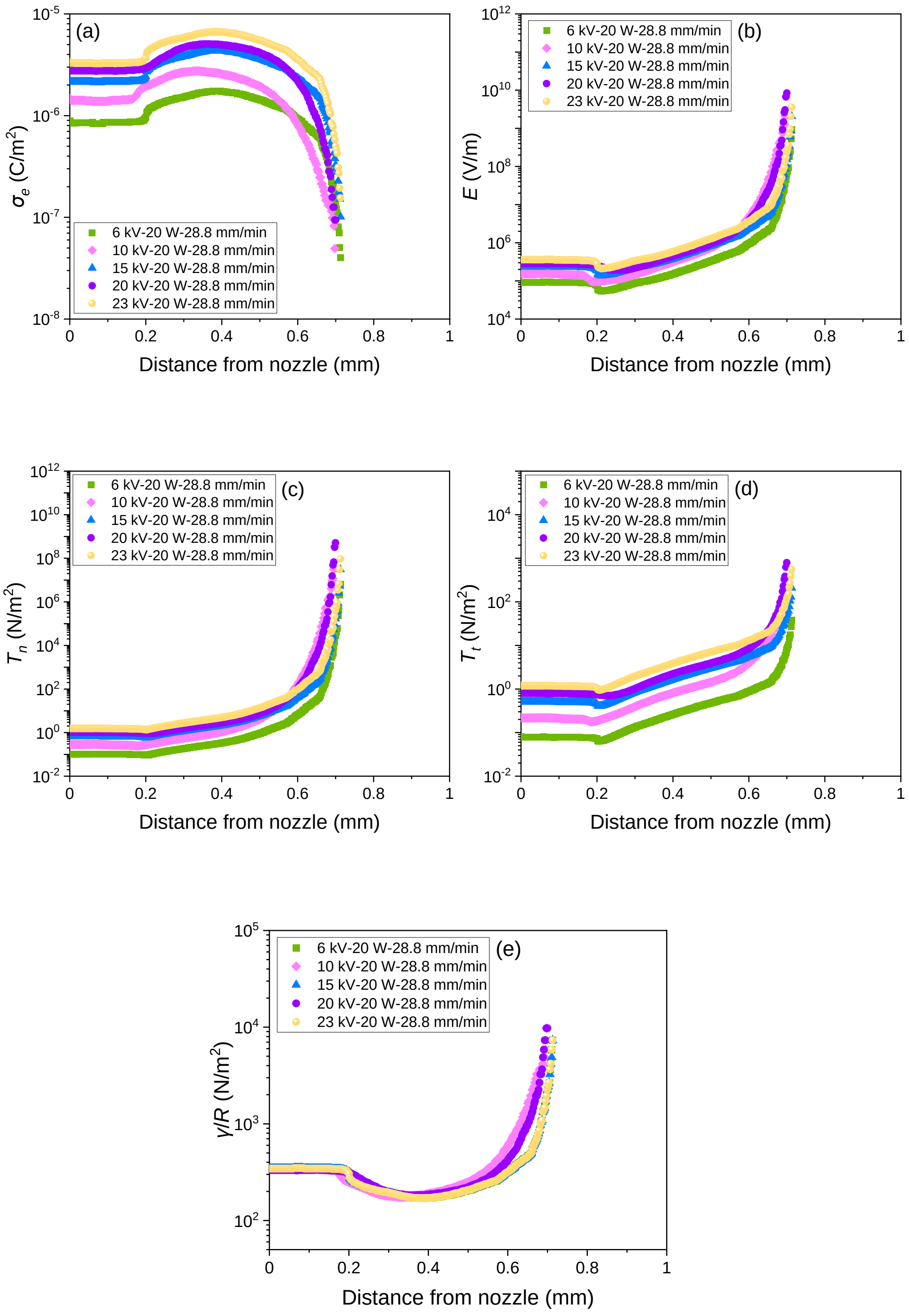


**Figure S5.** Effect of applied voltage on (a) charge density profiles, (b) electric field profiles, (c) normal stress profiles, (d) tangential stress profiles, and (e) cohesive force profiles for the feeding rate of 28.8 mm/min and laser power of 20 W.

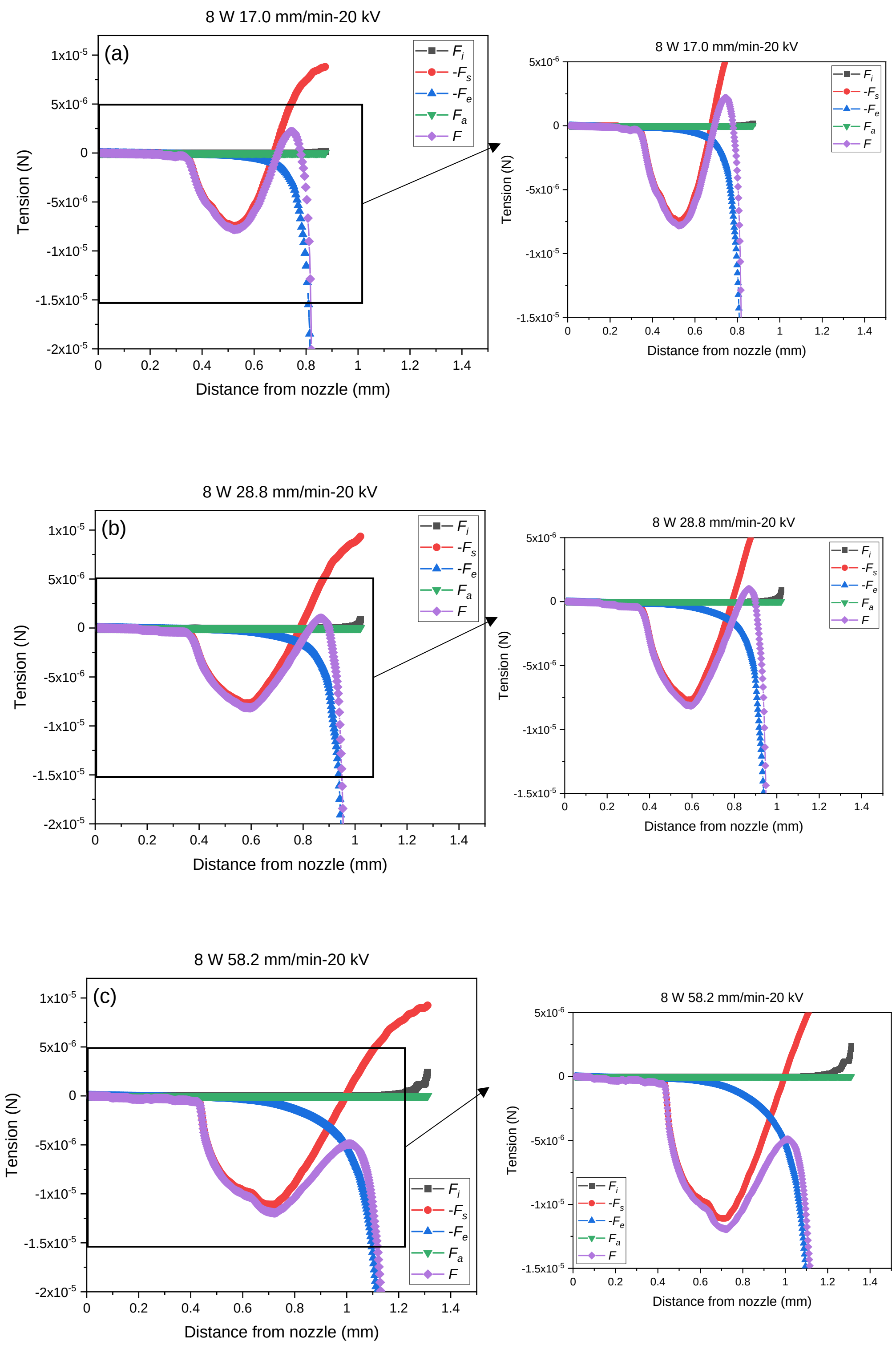


**Figure S6.** Tension profiles for conditions with the feeding rate of (a) 17.0, (b) 28.8, and (c) 58.2 mm/min, applied voltage of 20 kV and laser power of 8 W.

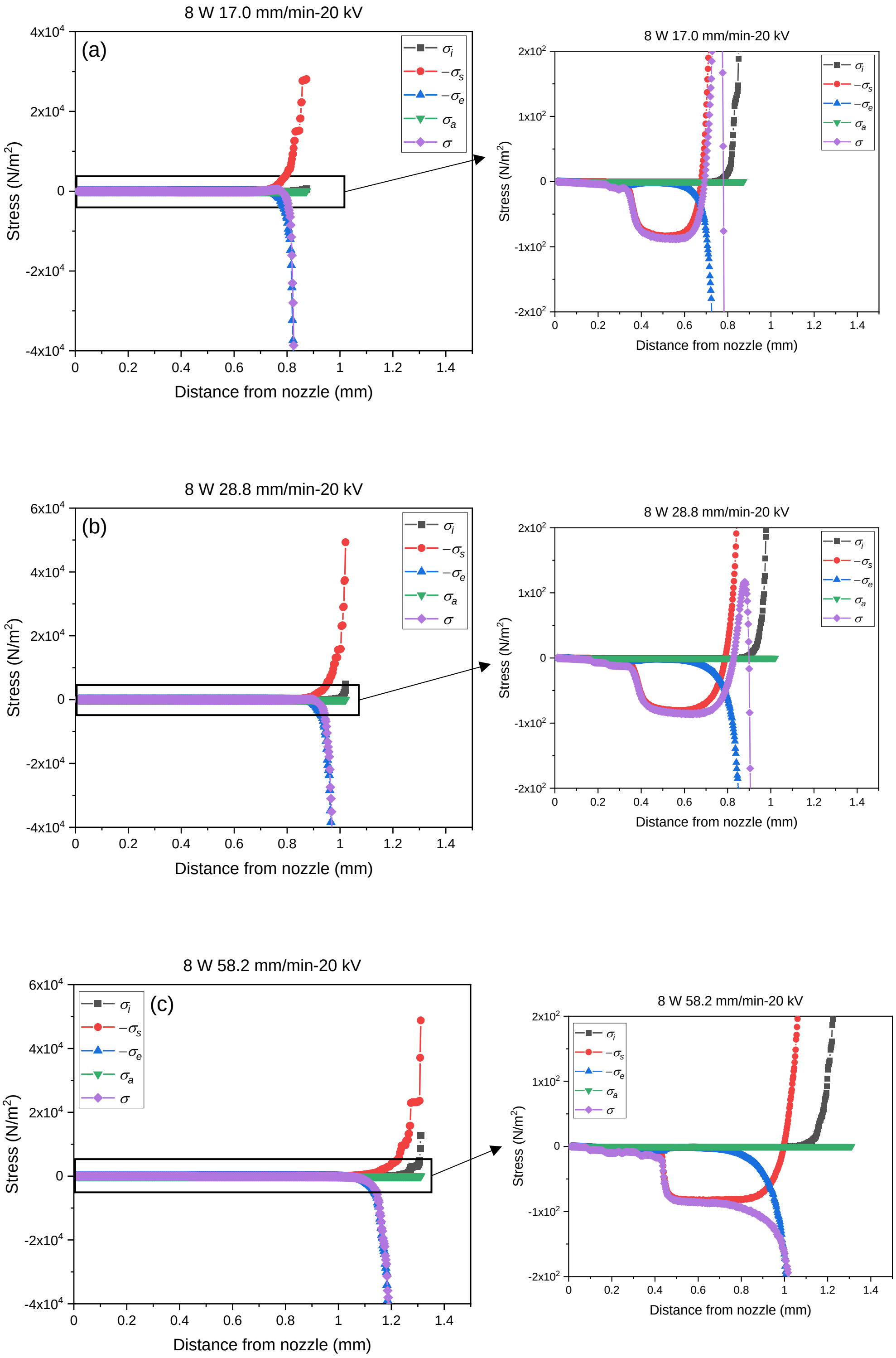


**Figure S7.** Stress profiles for conditions with the feeding rate of (a) 17.0, (b) 28.8, and (c) 58.2 mm/min, applied voltage of 20 kV and laser power of 8 W.

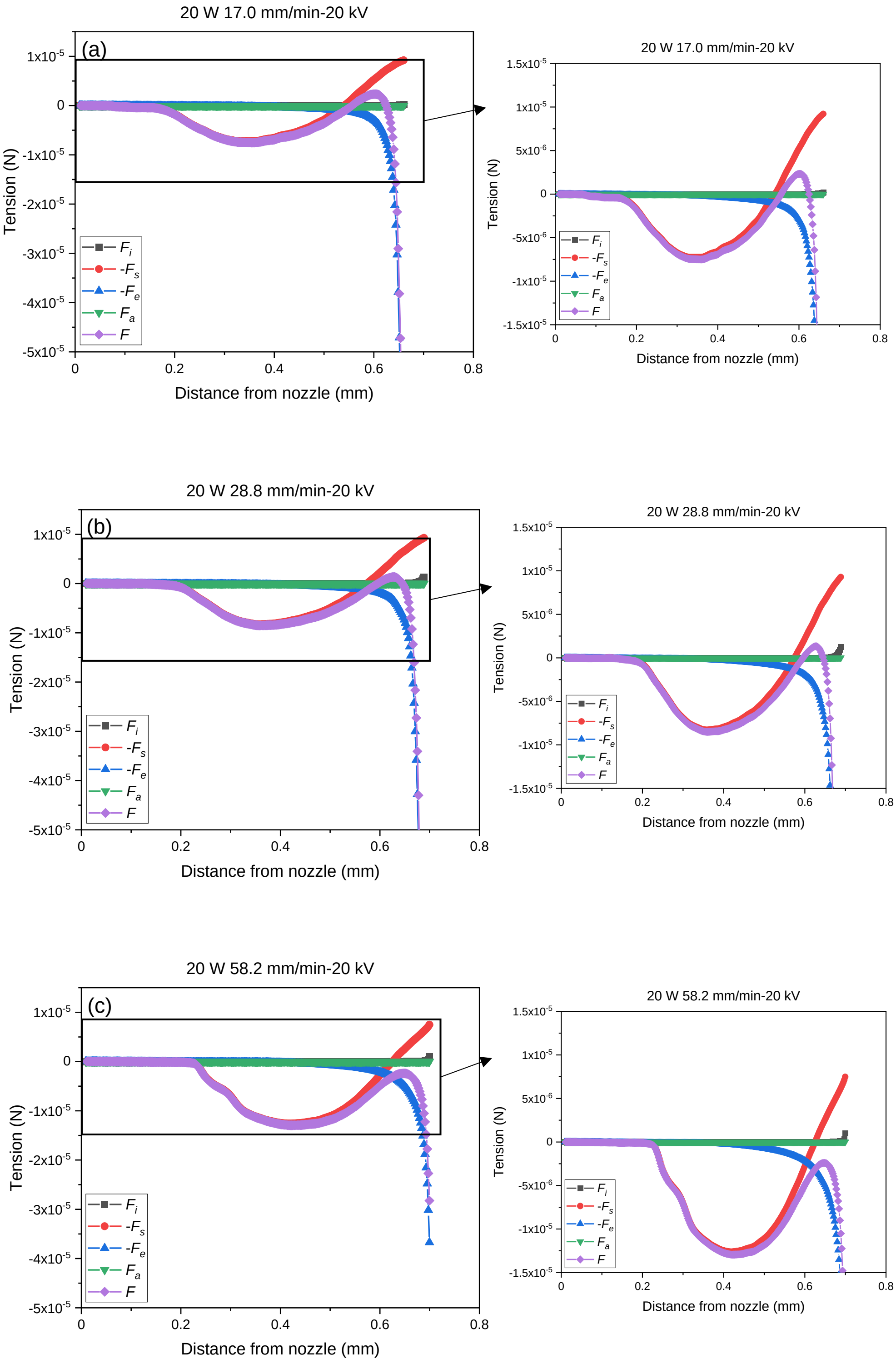


**Figure S8.** Tension profiles for conditions with the feeding rate of (a) 17.0, (b) 28.8, and (c) 58.2 mm/min, applied voltage of 20 kV and laser power of 20 W.

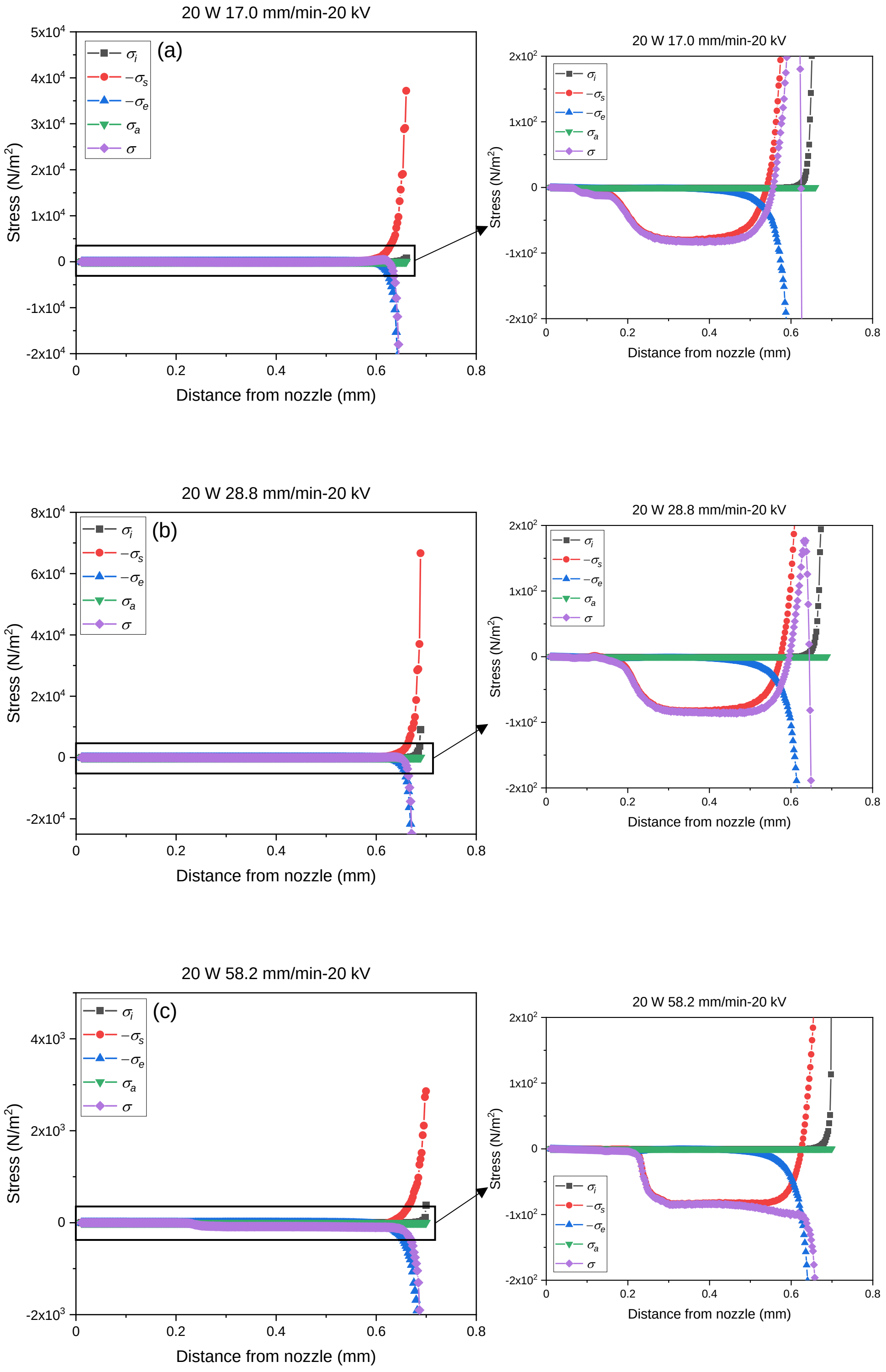


**Figure S9.** Stress profiles for conditions with the feeding rate of (a) 17.0, (b) 28.8, and (c) 58.2 mm/min, applied voltage of 20 kV and laser power of 20 W.

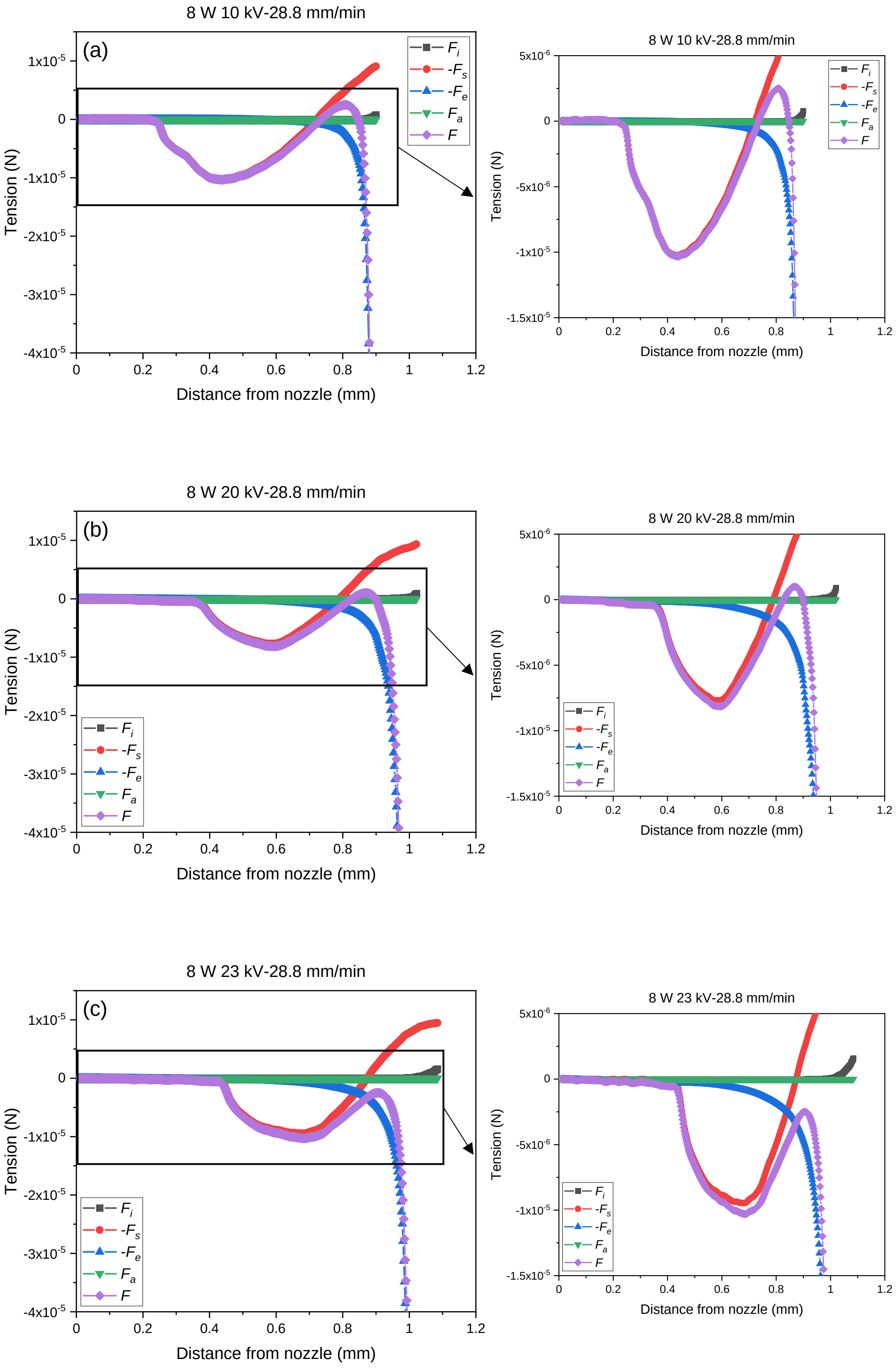


**Figure S10.** Tension profiles for conditions with the applied voltage of (a) 10, (b) 20, and (c) 23 kV, feeding rate of 28.8 mm/min and laser power of 8 W.

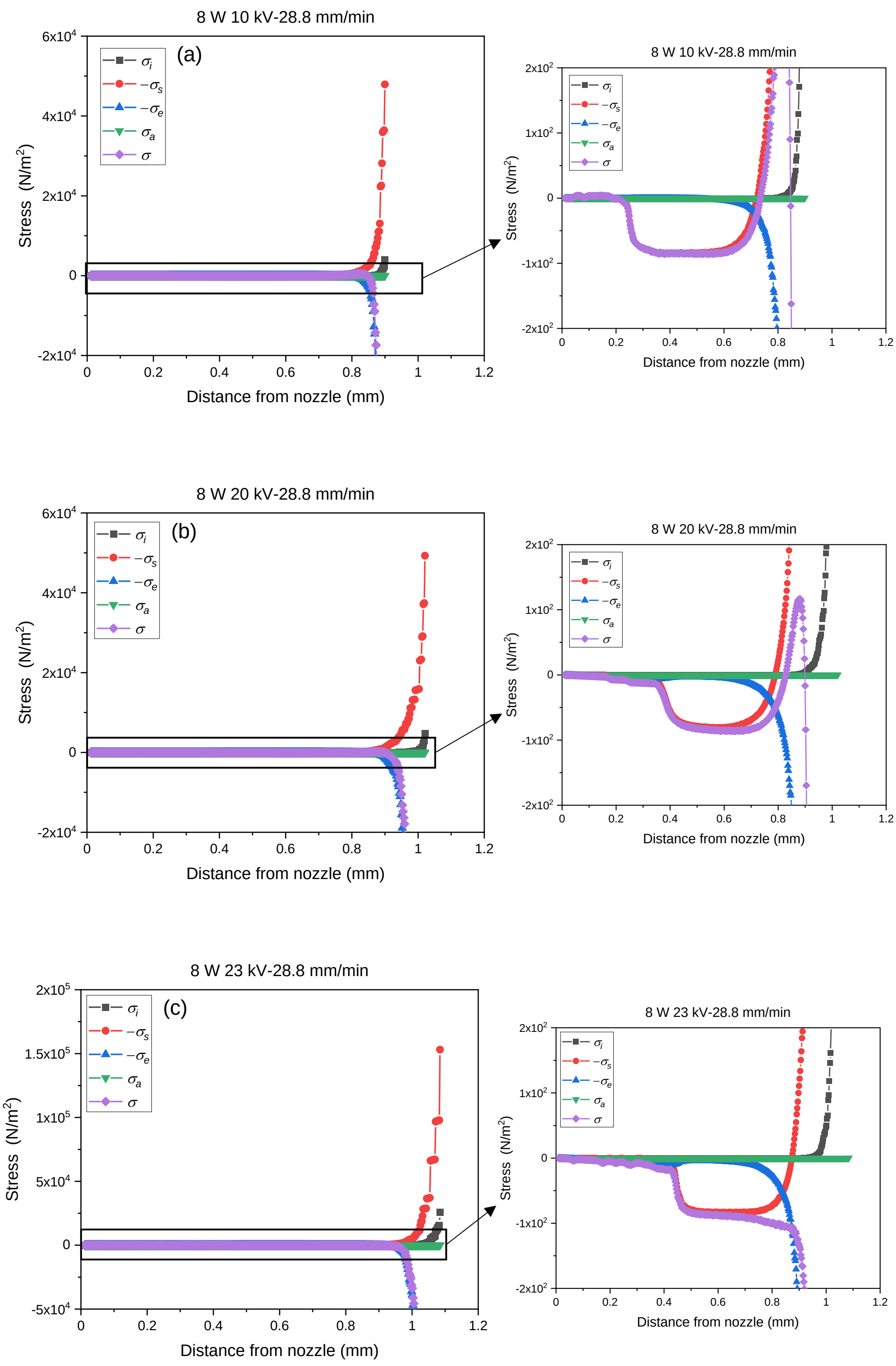


**Figure S11.** Stress profiles for conditions with the applied voltage of (a) 10, (b) 20, and (c) 23 kV, feeding rate of 28.8 mm/min and laser power of 8 W.

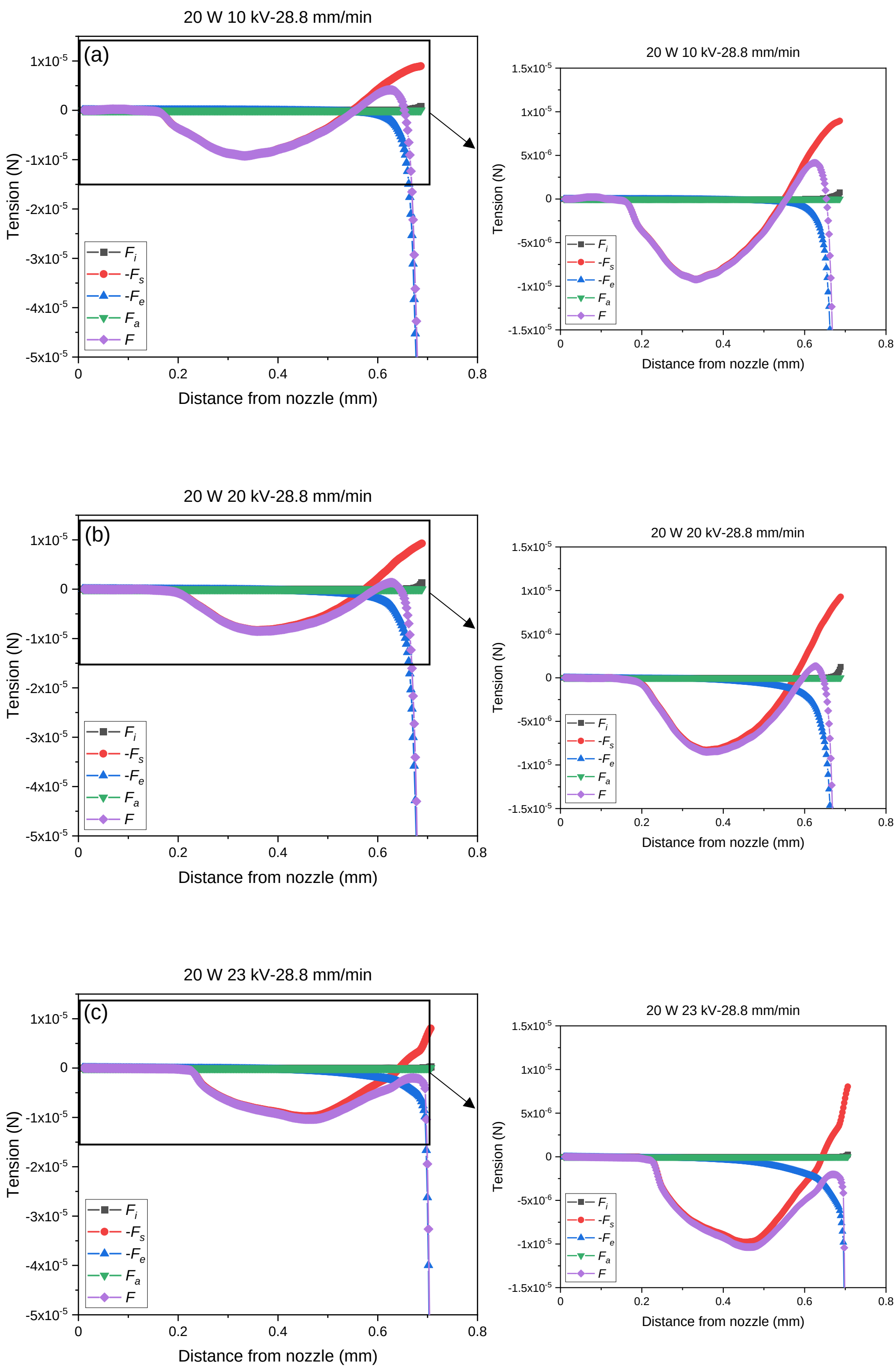


**Figure S12.** Tension profiles for conditions with the applied voltage of (a) 10, (b) 20, and (c) 23 kV, feeding rate of 28.8 mm/min and laser power of 20 W.

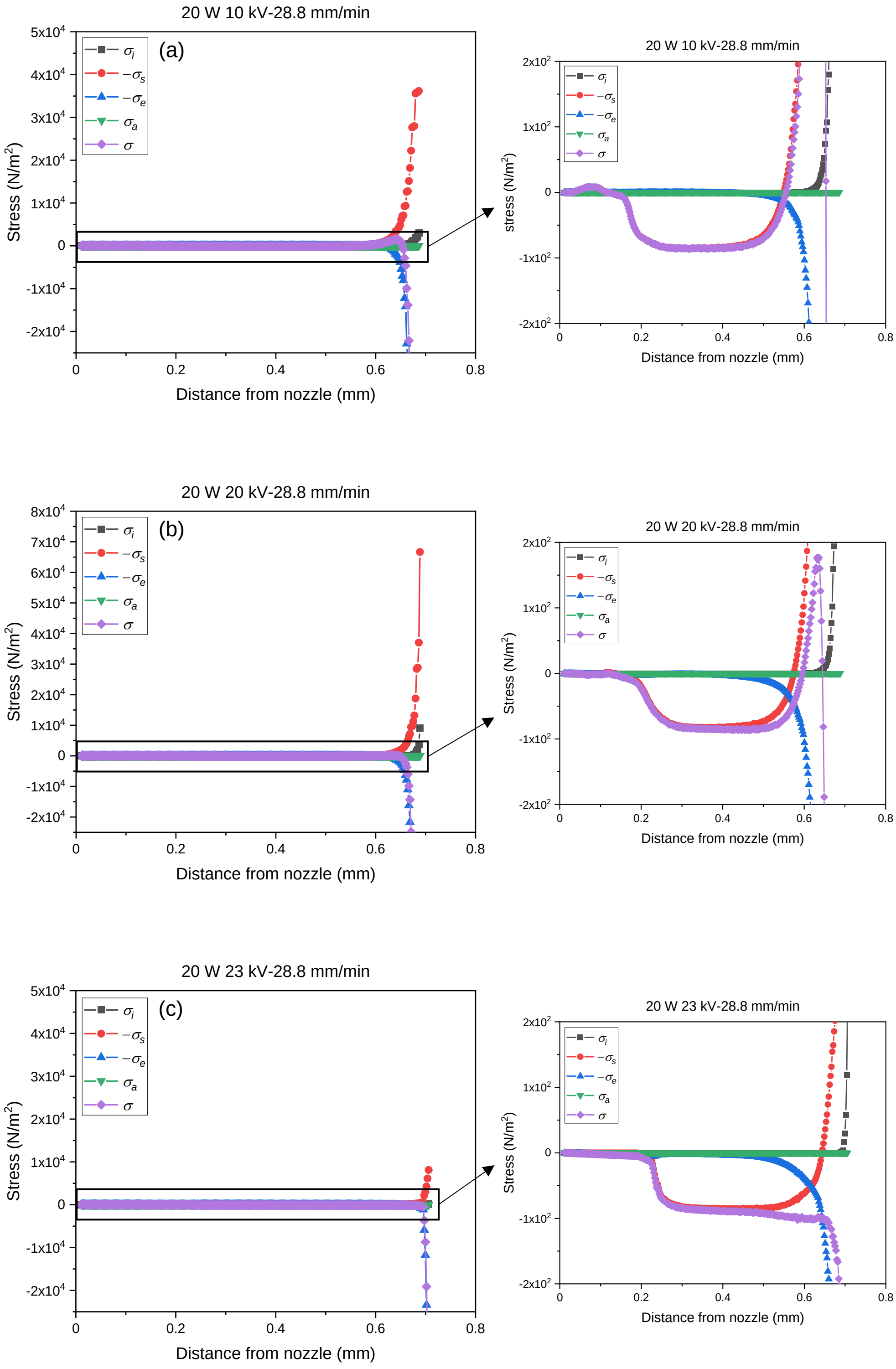


**Figure S13.** Stress profiles for conditions with the applied voltage of (a) 10, (b) 20, and (c) 23 kV, feeding rate of 28.8 mm/min and laser power of 20 W.

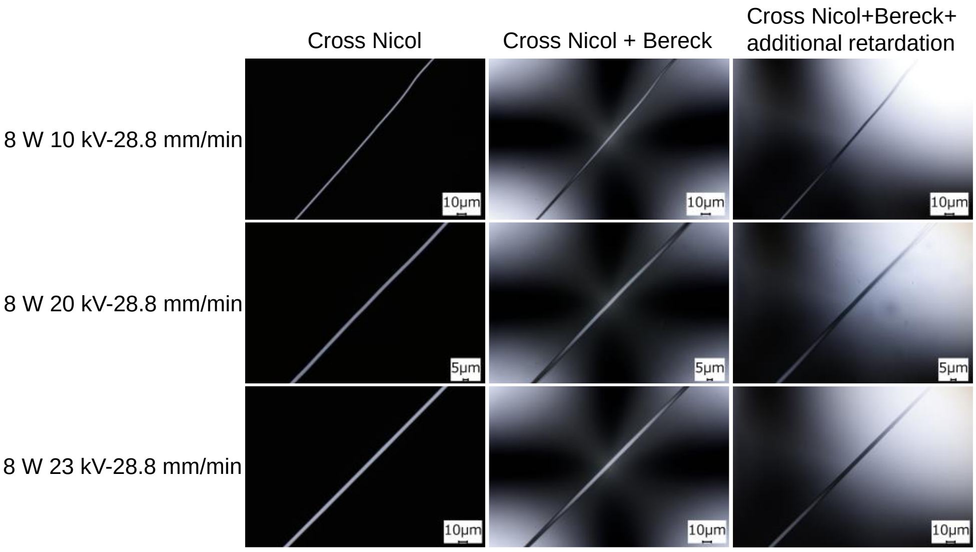


**Figure S14.** Micrographs of the fibers observed under a polarizing microscope: electrospun fibers for conditions with various applied voltage at laser power of 8 W. Cross Nicol, Cross Nicol + Bereck, and Cross Nicol + Bereck + additional retardation correspond to under cross-polarization condition, cross-polarization condition using the Bereck compensator without optical retardation, and using the Bereck compensator with optical retardation, respectively.
